# Patients’ healthcare, education, engagement, and empowerment rights’ framework: Patients’, caretakers’ and health care workers’ perspectives from Oromia, Ethiopia

**DOI:** 10.1371/journal.pone.0255390

**Published:** 2021-08-12

**Authors:** Zewdie Birhanu, Fira Abamecha, Nimona Berhanu, Tadesse Dukessa, Mesfin Beharu, Shimelis Legesse, Yohannes Kebede

**Affiliations:** 1 Department of Health, Behavior, and Society, Faculty of Public Health, Jimma University, Jimma, Oromia, Ethiopia; 2 Department of Pharmacy, Faculty of Health Sciences, Jimma University, Jimma, Oromia, Ethiopia; 3 Department of Internal Medicine, Faculty of Medical Sciences, Jimma University, Jimma, Oromia, Ethiopia; 4 Department of Nursing and Midwifery, Faculty of Public Health, Jimma University, Jimma, Oromia, Ethiopia; 5 Jimma University Medical Center, Jimma University, Jimma, Oromia, Ethiopia; Drexel University, UNITED STATES

## Abstract

**Background:**

Successful health care and clinical services essentially depend on patients’ realization of ones’ rights, and health workers’ and facilities’ fulfillments and protections of these rights. However, little is documented about how patients and health workers perceive patients’ rights during care-seeking practices.

**Methods:**

A qualitative study was conducted in four hospitals in Ethiopia through 8 focus group discussions with patients and 14 individual interviews with diverse groups of patients, caretakers, and 14 interviews with health workers. Participants were recruited through a purposive sampling method to meet the saturation of ideas about patients’ rights. The sampled patients, caretakers, and professionals were enlisted from various departments in the hospitals. The data analysis was assisted by ATLAS.ti 7.1.4

**Results:**

The study identified three major categories of healthcare rights (clinical, socio-cultural, and organizational), incorporating supporting elements of education, engagement, and empowerment. Study participants reported detailed rights the patients would have during hospital visits which included the right to timely access to care and treatment, adequate medications) with full respect, dignity, and without any discrimination. Patients widely perceived that they had the right to tell their illness history and know their illness in the language they can understand. It was also widely agreed that patients have the right to be educated and guided to make informed choices of services, procedures, and medications. Additionally, patients reported that they had the right to be accompanied by caretakers together with the right to use facilities and resources and get instructions on how to utilize these resources, the right to be protected from exposure to infections and unsafe conditions in hospitals, right to get a diet of their preference, and right to referral for further care. Nevertheless, there was a common concern among patients and caretakers that these rights were mostly non-existent in practice which were due to barriers related to patients (fear of consequence; a sense of dependency, feeling of powerlessness, perceptions of low medical literacy), health workers (negligence, lack of awareness and recognition of patient rights, undermining patients), and facilities’ readiness and support, including lack of guiding framework.

**Conclusions:**

Perceived patients’ rights in the context of hospital visits were profoundly numerous, ranging from the right to access clinical and non-clinical services that are humanely respectful, fulfilling socio-cultural contexts, and in a manner that is organizationally coordinated. Nonetheless, the rights were not largely realized and fulfilled. Engaging, educating, and empowering patients, caretakers, and health care providers supported with policy framework could help to move towards patient-centered and right-based healthcare whereby patients’ rights are protected and fulfilled in such resource-limited settings.

## Introduction

The Universal Declaration of Human Rights (UDHRs) formalized in 1948 recognizes “the inherent dignity” and the “equal and inalienable rights of all members of the human family” [1]. A right-based and patient-centered health care was developed based on this concept of the fundamental dignity and equality of all human beings [2]. In its article 25, the UDHRs states about the rights to adequate health-i.e. everyone has the right to a standard of living adequate for the health and well-being of himself and of his family, including food, clothing, housing, and medical care. Moreover, article 12 of the International Covenant on Economic, Social and Cultural Rights (ICESCR) defines the right to adequate health relatively: the right of everyone to the enjoyment of the highest attainable standard of physical and mental health [1]. Patients’ rights vary in different countries and contexts depending upon existing socio-cultural and economic factors. The particular rights to which patients are entitled depend on the nature of the patient-physician relationship and are also influenced by the socio-political system of the countries [2]. Despite a continued debate on the topic, there is growing international consensus that all patients have a fundamental right to privacy, to the confidentiality of their medical information, to consent to or to refuse treatment, and to be informed about the relevant risk to them of medical procedures [2, 3].

It has been widely recognized that the quality of health care depends on the premise that optimal health can be best achieved in the context of active and well-informed participation of patients in their care and management [4, 5]. Participatory clinical consultations and informed decision-making is possible when patients are well aware and fully exercise their rights during health care seeking in general [2, 5]. A patient who knows his/her right can clearly and openly communicate about his/her health needs and expectations, facilitate participatory decision making, thereby reducing medical errors arising from miscommunication between health workers, patients, and families, and enhance trust and the therapeutic relationship [2, 3]. Moreover, the right-based approach to healthcare would promote the realization, fulfillment, and protection of patients’ rights as human beings, including dignity autonomy, equality and respect, participation and interdependence, and fairness in treatment throughout accessing healthcare during hospital visits. Therefore, the right-based approach mandates the health system in resource-limited settings to set health policy and delivery strategies in striving towards achieving a minimum standard of available, accessible, acceptable, and quality health and patient care that would, in turn, call to the involvement of stakeholders, the government bodies and other agencies to that end. World Health Organization (WHO) recommends a right-based approach to health to meet the needs of those who are left furthest behind towards greater equity and universal health coverage [6–10]. Therefore; it is vital to respect the right of patients to fully understand patients’ bio-psychosocial needs and problems in healthcare in general and every clinical encounter, in particular [2]. Thus, upon their visits to hospitals, patients need to know their rights while accessing the desired care and services, and when diagnosed, they need to develop an appropriate understanding of their condition through clear explanations, seeing treatment demonstrations, and have ongoing support to learn practical skills needed to manage and control their condition [2–4]. These outcomes are found to be achieved via patient-provider-centered care where providers also recognize patients’ right to right-based care provision that is consistent with principles values, needs, and desires of patients [2, 3, 5].

In Ethiopia, health services are limited and of poor quality relative to other low-income countries [11, 12]. A wealth of literature documented that patient dissatisfaction remains high in the country and a major challenge to the Ethiopian care delivery system [13–20]. Despite the tremendous efforts given to expanding access to health facilities and the health workforce, the level of attention given to patients’ right and right-based approaches to healthcare is considerably low in Ethiopia [21]. In resource-poor settings, where patients are expected to face a multitude of challenges to demand and realize their rights regarding healthcare, it is crucial to assess how patients perceive their rights during health facility visits which can help to establish a favorable guiding framework to reinforce the practice of patients’ rights. This would enable us to assure the responsiveness of service delivery to ensure and safeguard the rights of patients to access adequate and timely care with all due respect and dignity [2]. In Ethiopia, health is a constitutional right, clearly stated in the Ethiopian constitution and the patient has the right to access medical care without any discrimination and barriers [22]. Despite constitutional recognition, until recent times, the issue of patients’ rights has not been given due attention in terms of realization and fulfillment in Ethiopia. And, more importantly, no elaborate legal, policy, and regulatory frameworks that protect and safeguard these rights exist in the country. The recent national health sector transformation plan has vowed to develop a legislative framework to reinforce compassionate and respectful care (CRC) which can include regulation on patients’ rights and responsibilities as well [21]. Few studies explored how patients and health care providers perceive patient rights during medical visits and these findings indicated that the awareness and recognition of patients’ rights were quite limited across settings [23–26]. This suggests that patient right often ignored in medical research and practice and it is rarely subjected to scientific inquiry, particularly in resource-limited settings including Ethiopia. Therefore, the objective of the study was to explore perceived patients’ rights during health facility visits for healthcare and medical consultations, from the perspectives of patients, caretakers, and health care providers. Moreover, the study was aimed to inform policy and the ongoing efforts to develop patients’ rights policy framework in Ethiopia and beyond.

## Methods and materials

### Study setting

The study was conducted during June 2019 in hospitals found in Jimma zone, Oromia region (the largest national regional state in Ethiopia). The zone is located at 352 km away from Addis Ababa/Finfinne (the capital city of Ethiopia). Three of the hospitals (Shenen Gibe, Agaro, and Seka Chokorsa) were district hospitals and one was serving as a referral and teaching hospital (Jimma University Medical Center). All the hospitals provided a wide range of health services/cares at preventive, outpatient, and inpatient departments and were purposively selected to represent a diversity of inpatient perspectives and views on the study objectives.

### Study design

The study used a qualitative approach guided by the grounded theory process to understand patients’ and health care providers’ perspectives on the patient’s rights while seeking care in the hospital. Grounded theory was preferred as it is the most useful approach to inductively develop key elements and dimensions that are grounded in systematically gathered and analyzed data [27].

### Population and sampling

The study population included purposively selected patients (who were able to actively participate in the study) and caretakers (i.e. person who assisted or accompanied patient) who visited the selected hospitals during the study period. The patients were included from across different wards (in-patient, out-patient, emergency, surgical, internal medicine) and clinics (chronic diseases, and maternal and child health). Gender and residency in rural/urban were also considered for inclusion. In addition, health workers from different professional categories were included. Overall the selection of participants and data sources was made stepwise to meet the purpose of saturating ideas about patients’ rights. Theoretical sampling (i.e. simultaneously collecting and coding the data to decide what data were to be collected next) was used to construct dimensions and develop a right-based patient care framework. Throughout sampling, we excluded patients who were severely ill and unable to respond.

### Data collection methods and procedures

The data were collected through focus group discussions (FGDs), in-depth interviews, and key informant interviews with patients, caretakers, and health staff. Prior to the commencement of data collection, the selected health facilities were contacted to get permission to undertake the study. However, study participants were contacted only at the time of the interview or discussion. All the interviews and discussions were conducted in the hospital settings and besides the participants and data collectors, no other person was present at the time of interviews and discussions. A pre-tested open-ended discussion/interview guide was used to facilitate the discussion and interviews. All FDs (ranged from 60 minutes to 130 minutes) and interviews (ranged from 40 minutes to 71 minutes) were recorded using a digital voice recorder besides note-taking during each discussion and interview. Saturations of ideas have guided the determination of the size of the interviewee and FGDs. The data were collected on patients ‘/caretakers’ and health workers’ perceptions and experiences concerning patients’ rights. Perception is how patients’ rights concerning care, service, and supports in the hospital facilities were sought to be conceived and understood by the participants. Rights in the current context are anything that is perceived as basic requirements to be realized, fulfilled, and protected for patients visiting hospitals for healthcare.

### Focus group discussions (FGDs)

A total of 8 FGDs were conducted (each 4 with males and females, separately). Two FGDs were conducted per the hospitals. The FGDs were conducted with diverse groups of patients and caretakers. Each FGD was conducted by two trained public health master-level experts with relevant experience in qualitative techniques and fluent in local languages (Afan Oromo and Amharic language). A team of investigators (all males and health professionals by training with master and above qualifications) with rich experience in qualitative researchers closely supervised the data generation process and also concurrently engaged in a review of transcripts to ensure quality and direct further data collection. In each FGD, the participants ranged from 7 to 11. Patients were selected for FGD through a purposive sampling approach to ensure diversity in age and service delivery departments which included different wards such as delivery, outpatients, and inpatient mixes, obstetrics and gynecology, surgical words, emergency ward, inpatient department, and medical words wards. Moreover, the participants were balanced with patients and caretakers to capture diverse views and experiences during hospital visits. The discussions were conducted in a private room where the privacy and comfort of the participants were kept. However, at district hospitals, few discussions were held within the hospital compound under a tree.

### In-depth interviews with patients

In-depth interviews were conducted with a total of 14 patients/caretakers from the four hospitals included in the study. Five patients were sampled from JUMC and nine patients/caretakers were sampled from the district hospital (3 in each). Patients were sampled to ensure diversity in age (ranged from 20 to 75 years), sex (6 females and 8 males), and diverse service delivery points which included medical wards, gynecology, and obstetrics, pediatric clinics, inpatient service, outpatient department (adult and pediatric). Moreover, four of the interviewees were caretakers of the patients. The caretakers were included for two main reasons: replacement, when the selected patients were unable to express their ideas and were not fully aware of their experience because of sickness; and interactions, the experiences they had with the hospital system in general and with health workers and other hospital community in particular. This enabled us to capture a wide range of experiences as caretakers of the patients were frequently supporting and interacting with hospital staff and health workers. In pediatric clinics, caretakers of the children were interviewed. Most of the interviews were conducted by two public health experts, and one interview was conducted in Kefegna language by one Ph.D. student and transcribed verbatim to English. The interviews were conducted in a private setting to ensure the privacy and comfort of the patients.

### Key informant interviews-KII (in-depth interview with health staff)

To triangulate the findings of interviews and FGDs from patients, a total of 14 key informants (mostly health workers) were interviewed with purposively selected staff, at least two key informants from each Hospital. Consequently, diverse groups of health care providers including general practitioners (GP), medical interns (in JUMC), nurses, pharmacists, laboratory technologists, and administrative staff (human resource and hospital guard) were included in the key informant interviews. To ensure the diversity of experience, health care providers were also purposively selected considering the durations of their experiences. The KIIs were also conducted by trained master-level qualitative researchers. The data collectors were recruited only to conduct and transcribe the discussions and interviews and they did not have any interest in the study.

### Data analysis

The data from FGDs and interviews were transcribed verbatim and then translated into the English language for analysis. The coding and further analysis of the data was assisted by ATLAS.ti 7.1.4. The transcripts were read and re-read by investigators who then assigned codes (open coding) and came up with an initial coding structure. To ensure the relevance and appropriateness of the coding structure, one coder (ZB) (CV is shown in S1 Appendix) undertaken iterative rounds of open coding of selected transcripts guided by the grounded theory approach and the second coder (YK) (CV is shown in S2 Appendix) reviewed and verified the emergent codes. Finally, the research team reviewed the coded transcripts and generated codes to reach a consensus on the coding system and consistent code definitions used to code all transcripts. The initial list of code families and their members is shown in S3 Appendix. Results were organized in themes and sub-themes elaborated by key quotes. The major themes and sub-themes that were emerged were summarized as a patients’ right to healthcare during hospital visits framework. Peer debriefings were conducted with the research team to inform interpretation. The consolidated criteria for reporting qualitative research (COREQ) [28] were followed in the design, analysis, and report of the manuscript (information is shown in S4 Appendix).

### Quality control

To ensure the trustworthiness of the findings, data were collected by experts in qualitative researches that were trained. The appropriateness and relevance of the guides were ensured through expert reviews and pre-testing of tools. The field team held a debriefing session each day during the entire fieldwork to guide the selection of further samples toward the richness of the data. Facilitators’ and note-takers impressions were documented for each data source. Data from FGDs and KIIs were triangulated to ensure credibility. Representations of primary and referral hospitals from across different settings were made to enhance the transferability of the findings.

### Ethical considerations

This study was ethically reviewed and approved by the Institutional review board of the Institute of Health, Jimma University (Ref. No: IHRPGD/669/2019). All study participants were explained about the purpose of the study and informed verbal consent was obtained from each respondent.

## Results

### Participants’ demographic profile

Except for two respondents who dropped out from the FGDs due to reasons related to service provisions, all invited people successfully participated. In male FGDs (four FGDs), 33 patients/caretakers (8–9 per group) participated and the average age was 41.8. Thirteen participants were recruited from the outpatient department and the remaining were from different wards (inpatient departments). In terms of education, 7 participants had no formal education and the remaining attended primary and secondary schools. For female FGDs (4 FGDs), 34 patients/caretakers (7–11 per group) were part of the discussions with an average age of 30.0 years. In contrast to male FGDs, 19 participants in female FGDs were not attended school; about 12 were recruited from the outpatient department whereas the remaining was sampled from different medical wards (inpatient departments). On the other hand, 14 patients (6 females and 8 males, 9 from the outpatient department and 5 from inpatient services) eight with no formal school participated in the in-depth interviews. The age of those patients ranged from 20–75 years (average 34.9). Likewise, 14 health staff with diverse professional and career backgrounds (3 GPs, 4 Nurses, 2 laboratory staff, 1 psychiatrist, 1 human resource administrator, and 1 hospital security) were involved in the in-depth interviews.

### Perceived rights of patients during hospital visits

**Fig 1:**
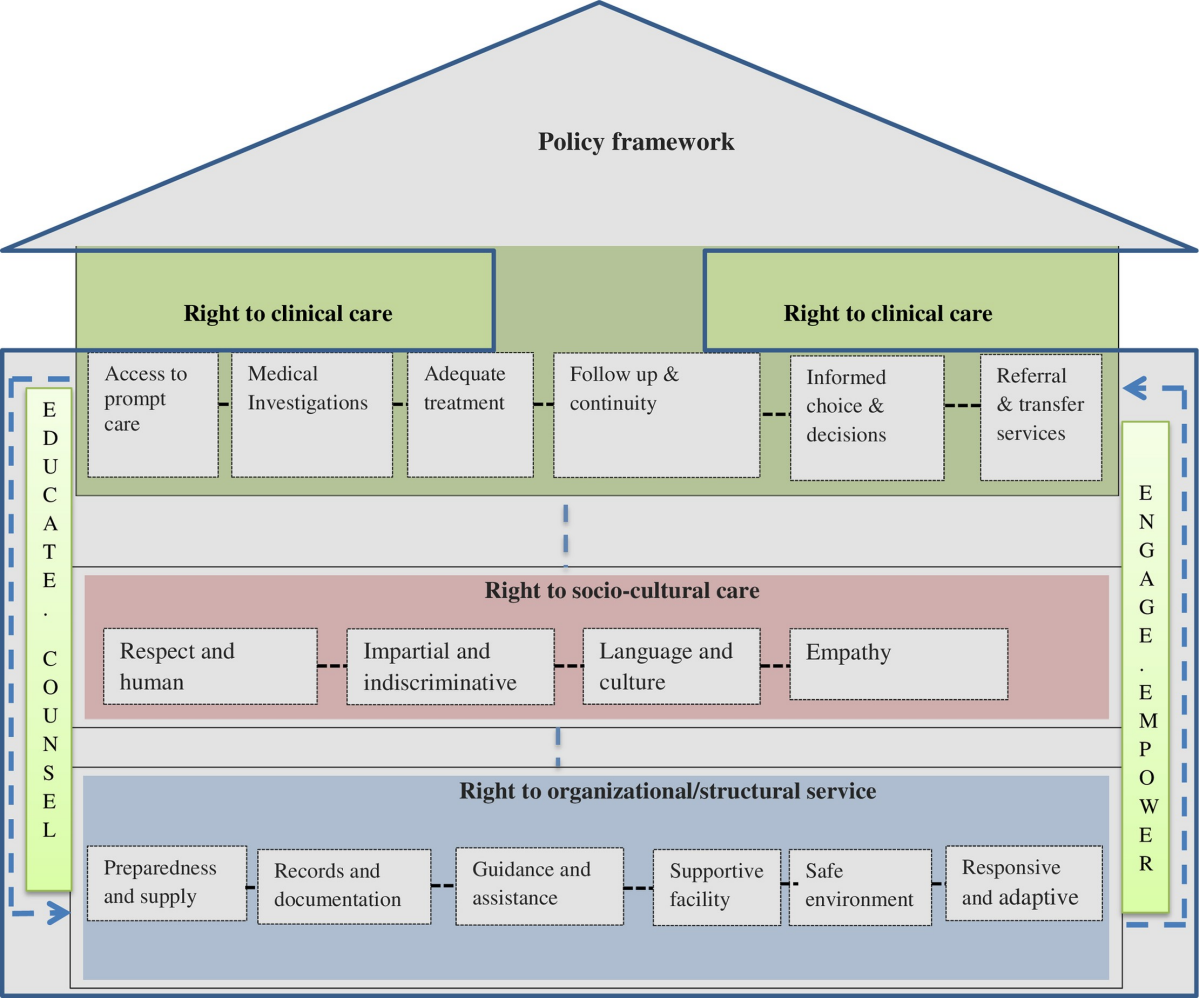
An emergent framework of perceived patients’ rights in the healthcare process in the study contexts, Oromia, Ethiopia, June 2019. As emerged from data through inductive analysis, there were three major dimensions of patients’ perceived rights to adequate healthcare which included organizational or structural dimension, socio-cultural aspect of care, and clinical care. Each dimension consisted of more specific rights conceptualized and presented according to hypothesized relationships to each other that suggest the potential pathways to enhance patients’ rights in the healthcare delivery process. In this conceptualized framework, education, counseling, engaging, and empowering patients were emerged as cross-cutting and supportive to the fulfillment of other components of patient’s rights. Additionally, devising a policy framework on the patient’s right to healthcare provides an institutionalized platform to protect patients’ rights. The details of the major perceived healthcare rights with sub-categories were described below.

The participated patients and healthcare workers revealed a wide range of patients’ rights during health facility visits for adequate healthcare. Fig 1 depicts the emergent framework.

### Rights to clinical care

Patients’ rights to technical care during hospital visits for healthcare were made up of essential rights that serve patients to access an informed choice to quality medical diagnosis, treatment, and follow-up services.

#### Right to access treatment

This right embraces access to services of all forms at any time of visit without compromising quality. All categories of study participants indicated that the ultimate purpose of patients visiting hospitals is to get treatment and they overtly stated that patients have to right to get the service, care, and treatment they need without any barriers, discrimination, and language barriers. Terms such as “enough treatment”, “adequate treatment”, “get services any time, 24 hours in a day”; free services as per indicated in-laws and regulations (eg for poor and who cannot pay), “get treated on time”, “well treated” were used mostly by patients to describe this particular rights. This included the right to get a bed for accommodations.

*Our clients have a right to get any services on time; they have a right to receive services at any time in 24 hours*.—(KII, Health worker)

#### Right to medical diagnosis and investigations

This right includes access to quality and prompt investigations. Particularly, FGD participants and key informants explained that patients have the right to access laboratory services and investigations and the right to undergone necessary physical examination based on the consent and willingness of patients. This right also included the right to be well informed about procedures and investigations and refuse to give samples if they don’t want to.

*A patient has the right to be treated*, *the right to have a necessary examination and have medication*, *the right to be respected and treated well*.- FGD_Male (30-year-old, caretaker)

#### Right to quality and prompt treatments

There were significant reports concerning rights to access to drugs and medications within the fastest possible timeframe: Views from all categories of participants indicated that patients have full right to get necessary and efficacious drugs and medications with appropriate dosage. And it was ascertained that patients have the right to choose officious drugs and the right to refuse to take any drug as well. Uncooperativeness of pharmacists/dispensers and lack of drugs within hospitals are reported to have affected patients’ right to access drugs.

*They refused me the drug that is available here*. *He collected my prescription paper*, *but he was neither returned my paper nor gave me a drug*. *Then*, *left them and bought it from a private pharmacy*.—FGD-Female (47-year-old, patient).

Promptness of care was an important aspect of rights to effective treatment as many respondents indicated that a patient has the right to be seen on time, and get prompt care and medication. Delays in laboratory and investigation results critically challenged the promptness of treatments of illnesses. Patients complain about this aspect of right mainly due to delays in laboratory results.

*My doctor ordered me for a stool examination and I gave my sample*. *The laboratory person told me that the result of the examination will be sent to the doctor*. *On the next day*, *my doctor has asked me for the result and I told him that I have given the specimen and they told me the result will be sent to my ward*. *When I went to the laboratory room it was closed*. *I think that it is not open on weekend*. *Today is my third day*. *There is no result yet and I am in pain*.*—*FGD-Female (40-year-old, patient).

#### Right to follow-up care and continuity

Right to get an appointment or follow-up care. The loss of patient files, restarting the entire treatment process as a new patient, and access to caretakers who initially treated the patients were common challenges to the continuity of care.

*They have the right to get an appointment to follow up and come on time by appointment date and utilize their care*.—KII 11(Health worker)

Inherent to the right to continuity of care, there was the right to be treated by the same doctor or provider and the right to know their disease: Patients concerned that was the frequent change of doctor who initiated their treatment. They mentioned that the provider who initiated their treatment process was often replaced by another on next the day. Concerning this, there was a consistent report that patients have the right to know and get explanations and information about their illnesses or diseases in the language they would understand. Many patients replied, “I have to learn what my illness is”. Regarding the frequent change of doctor, FGD participant said,

*The first doctor who diagnosed you couldn’t be available next time. Since they are being changed every time, they speak differently. They don’t tell you about your illness. For example, since I have known his sickness at the health center, they didn’t tell him about his illness. He came here because of his illness. How could he know his internal illness unless they make a diagnosis and tell us what it is! They didn’t tell your illness.—FGD-Female patient.*

There was another important aspect of continuity of care i.e. the right to get the treatment that is carefully monitored as follow-up progresses particularly in inpatient wards. ate: According to participants, the patient has the right to get continuous monitoring of their health progress and get updates and information. This also included the right to receive treatment adjustments based on patients’ progress.

A patient who denied this right explained,

*The wound is not cleaned for four days*. *At an earlier time*, *they would clean the wound daily*. *We asked them and they said that they were not assigned there [medical ward]*.*—*FGD-18-year-old female

#### Right to get empowered, counseling, and information

Many participants (all categories) ascertained that every patient has the right to get education, information, advice, and counseling services as a vital component of health services. This included the right to get an education on self-care, medications, health challenges, health conditions, illness, service delivery flows, do’s, and don’ts in the language they understood.

*A patient who comes to this hospital has the right to get educational services on their health conditions and health challenges*.*34 year*.*—Old*, *female caretaker*.

Another 33 year male patient also said

*Knowing my illness is important to me. They should tell me about my illness and give me information about it. They should give me an advice*.

#### Right to informed decisions and treatment choice

Participants reflected that patients have the right to choose and make an informed decision about their care and have the right to choose services, drugs, and medications they want; right to refuse to take any drug or medications if they felt so; right to refuse any procedure, investigations such as being x-rayed; right to refuse operations; right to select health care providers for consultations and operations; right to refuse giving any sample if felt so, and right to a diet of their preference when admitted.

*They do have the right to so no to sample requests which they do not want*. *We do not force for the examination which they have rejected*. *They have the right to refuse to be put under uncomfortable procedures during an examination*.- (KII, Health worker).

#### Right to referral and transfer

Right to be referred for higher care when necessary and the right to be transferred to another hospital of their preference was perceived as patients’ right.

*For example*, *some patients if they don’t get services or operation as quickly as they want*, *they request for transfer to another facility*. *They may not believe in the care or services which are provided here*. *In such cases*, *it is their right to get transfer to other places*. *But*, *the patient should have to give confirm the transfer by signing it*.*-* (KII, Health worker).

### Rights to socio-cultural oriented healthcare

Patients who visited hospitals unanimously underscored multiple social aspects (human-centered, empathetic, language, and culture-oriented, impartial, and focused on empowering) that the healthcare process should meet.

#### Right to get welcoming, respectful, and human-centered service

Patients expressed that disrespect was a concern to several patients and they underscored that they have the right to be respected. They argued that health workers and hospital staff including guards shall respect the patient’s dignity and morals in the whole process of seeking care and use of resources and facilities in the hospitals.

*As a patient, my rights are to be welcomed in a good manner. Welcoming is very important. It is a right for me.-33 year- old patient (IDI)*

*They [health workers] are disrespectful -nama ifatu-*, *they disregard you*.*-* FGD-Female, patient

#### Right to access empathetic care

This includes the right to tell illness stories and express feelings in the process of healthcare. According to participants, patients have the full right to tell their illness history in their language and the right to express their feelings related to their illness.

*The rights of patients are the right to tell his/her illness*. *They have the right to ask about their illness and get all the necessary information about his/her illness*. *-* 35-year-old, male patient

#### Right to get services in their language

Many patients explained that the language barrier was one of the critical obstacles to access service and care they need though they have the right to get services, explain their illness, and get information about their illness in their language without any bias and prejudice. A participant said,

*We have the right to be treated, the right to have a necessary examination and have medication, the right to be respected and treated well in the language we understand. Unless we speak in Amharic, they don’t reply to us. They don’t listen to us when we speak in Afan Oromo. It should be stopped! The patient has a right to be treated in his language. FGD-Patient-Male*

Another participant said

*I remember what happened to me last time*. *When I tried to talk in that language [Amharic] imperfectly -‘makolataf’-they ignored me and humiliated me*. (40-year-old, female caretaker)

#### Right to impartial care, equal treatment, and no-discrimination

This category reflected patients’ right to get services and equal treatment without any discrimination by religion, language, ethnic background, and any other social and demographic background. Another participant explained,

*I have the right to get fair treatments without any discrimination regardless of my language*, *religion*, *and other factors and expression of my feeling to the health care providers and giving suggestions*. (27-year- old, female patient)

Reflections from FGDs and in-depth (patients) interviews, however, patients lived experiences indicated that there were incidents of discrimination, especially by language.

*When we go out for purchasing drugs*, *the guard can’t allow us to re-enter the hospital*, *even if we showed them our hospital identity card*. *They don’t permit us; sometimes they selectively permit some people who had no even a card*. *They favor people with their language*. *For the last 14 days*, *I have faced this challenge many times*. *Today afternoon*, *I went out to purchase drugs for my sister who was to undergone an operation*, *but they didn’t allow me*. *I gave the drug to another person through-hole of the gate and they took it to the surgeon*. (FGD, 22-year-old, male caretaker)

### Rights to access favorable organizational and structural services

There were several rights mentioned by patients and health professionals about organizational arrangements required to ensure structural, logistics, physical facilities, and platforms in favor of respecting patients’ access to technical and socio-cultural rights and care.

#### Right to get served in responsive and adaptive facilities

Closely linked to the clinical and socio-cultural aspects of patients’ rights, both patients and health professionals claimed a lot of challenges that the hospitals as organized facilities were not responsive to while needing urgent responses and adaptive solutions. For example, the absence of adequate diagnostic tests, delays in laboratory results, lacks of respect, losses of patient files, and discontinuity of follow-up services were some. A female FGD participant said,

*“……When I went to the laboratory room it was closed. I think that it is not open on weekend. Today is my third day without getting my test results. There is no result yet and I am in pain*.

#### Rights to get adequately prepared and supplied hospitals

Patients’ access to quality care, treatment, and drugs was perceived to be unthinkable by respondents for which the hospitals should be prepared in terms of supply and logistics. A 35-year-old FGD participant explained,

*The drug is not found in the hospital, they [another patient] have been searching out all night across the town. If there is no drug found in this compound, please tell them to make it available*.

#### Right to documentation and access to personal medical records

Access to ones’ medical history was reported as a patients’ right. However, several patients complained that getting their medical cards is very difficult as a result of poorly organized documentation. There were occasions when patients’ files are lost, and investigations were restarted even for patients on follow-up.

*It is not easy to get a medical card*. *They do not accept us*. *There was a time when I didn’t get my card until 5*:*00 pm and spent the whole day without services*.*-* 28 years old, female FGD participant

#### Right to guidance, assistance, and communication

This includes the right to use facilities, resources, and right to get instructions, directions, assistance, communication or instructions on how to access and use them: Reflections from all categories of participants indicated that patients have the right to access and use resources and facilities available in the hospitals, especially water and toilets and has also the right to get education, guidance, support/help, and instructions on how to access and utilize these resources. In addition, it was indicated that they have to get support/help to locate service delivery points and directions for following appropriate care delivery flows in the hospitals.

*Ayi… some don’t know the consultation room or miss their ward or room*. *Therefore those who welcome patients [runners] should have to show the direction to patients*. *It is their responsibility as far as they are here*. *-* 21 year old caretaker

*Cleaners usually close the latrine during the nighttime*. *I don’t know where we have to go at a tight time*. *It is a complex building even to exit to go to the open field*. FGD-Female

#### Right to get conducive and supportive facilities and services

The patients ascertained that they have the right to be assisted by their families who can assist and support them during their stay. In the meantime, they added that it was their right to get conducive facilities and support for their caretakers, and families, and relatives. Some of the conducive services the participants claimed include access to resting places, appropriate food services, and hygiene facilities for caretakers. Patients also mentioned that takers s have the right to move freely in the hospital compound at any time. Regard this concern, a male FGD participant said,

*In this hospital, caretakers do not get a place, only patients do, caretakers are being bitten by mosquitoes and colds on a corridor. Therefore, it is better if a resting place for caretakers is prepared*.

Another FGD participant explained this issue as follows

*Some Guards are not flexible to listen to your concern. They are rigid and do not willing to listen to you. My relative brought food for me, but she was not able to enter the hospital. Such things should be corrected in the future. I begged the guards for one hour 1 hour but not possible. Also, these guards were throwing offensive words at me and I shouted at him not to say such naughty words.-30 year- old, patient*

Regarding food services, patients and health workers expressed that patients have the right to get a meal of their preference and recommended meal while on patient care.

*Before they took my history*, *they admitted me to the labor ward*. *Then*, *I stayed there for 24 hours without any food*. *On the next day*, *I have asked a doctor that I am dying from hunger*. *He replied to me that” aren’t you on labor”*? *“Do you need food*?*” he said*. *Then*, *I told him that I am not in labor*. *Then*, *he told to somebody else and they took me to another room and I got food and saved*.- FGD-Female.

#### Right to a safe physical environment that is not harmful to health or well-being in hospitals

Participant patients and health workers confirmed that patients have to tight to get protected from exposure to infections while staying in hospitals, and get access to preventive tools like mosquito nets. A FGD participant male patient said that;

*The patient should not be eaten by mosquitoes. We come here to be healed from our illness but acquiring another illness is wrong. This is our right but not respected*

*At night time I came here for asthma and they gave me glucose at that time*. *Then when I went to the toilet the next morning*, *my illness gets worse*. *It was not only the toilet but also the hospital environment that exacerbated my illness*.—(FGD, 30 years old, patient)

### Perceived barriers to patients’ right fulfillment

The study indicated that even though many patients had a good awareness of their rights that have to be protected, most of their rights remain unfulfilled. Different barriers were reported for the failures to fulfill patients’ rights during healthcare-seeking which explained as follows;

#### Fear of consequences

Some patients reported that due to fear of rejections and denial of services they didn’t speak out to demand their rights. Some patients believed that health workers may deny the service at all if they speak for their rights. Hence, counseling service is needed to encourage patients to fearlessly ask for the fulfillment of their rights.

*We get sick and our sickness gave them [HWs] all rights. If you tell them it is a government that assigned them to serve us [patients], they may expel us from the hospital [hospital compound]. Then we leave our home with our illness without getting any care. - 75 year- old patient (male)*

Because of fear of rejection and verbal insult for speaking out about their rights, patients often beg for services instead of asking for the fulfillment of their healthcare rights. A 75 years old man expressed his concern as follows:

*We have fear. We may go home with our illness. We have to beg (yoo sossobanne) them to get a tablet (drug). We are begging for our rights. When we say this tablet is expensive (Wud), they don’t accept us. They may throw us away. Then, don’t you think we have returned to our home with our illness? Look! It is difficult to argue with an educated person! We only get services by beginning them! “Jala gugguufnee kadhachuu”. If we don’t, we have to go home with our concerns*.

It was also noted that the hospitals and the health managers were neglectful about protecting patients’ rights and health workers tend to blame patients for becoming sick.

*There was negligence on these rights, “maaltu na dhibeetu jira”. …there was nobody who works for the sake of humanity. Some say that i is your disease and prevent it yourself! … here was a deficiency in respecting patients’ rights.- 33-year- old patient (male)*

#### Patients’ lack of awareness about healthcare rights

Significant number of respondents (both patients and health workers) stated though the majority of the study participants knew their rights there are still patients who did not know the healthcare rights while seeking medical care and clinical consultations. In addition, health professionals also did not inform or educate patients about their healthcare rights. A 33-year-old patient explained this phenomenon as:

*Such like thing does not exist. Patients do not know their rights. Health professionals are also not educating or informing patients of these rights. People should have to know or be educated on how to practice their rights and responsibility. This thing is necessary but not respected in this hospital*.

A respondent (32-year-old, patient) who were unaware of patients’ healthcare rights said,

*Unless you tell me after this interview, I don’t know about it. I would simply accept even a poisonous’ thing as far as they would say it is ok because I am a farmer. We would accept what they order. If I refuse their advice, they may expel me from the hospital. Then what can I do!. What would I say to those an educated (famous) one?*

#### Perceptions of being patient and a sense of dependency

Many respondents (patients) responded patients are dependent persons and they believed that a dependent person has no right to claim rather required to accept the advice prescribed by the health care providers. This finding indicated the need to empower patients so that they start to ask for their rights and engage in decision-making. A 35- year-old patient explained this issue as;

*R: Aaaahaa…….you brought a difficult question! Eeehhh……if I tell them ‘this is my right and do this and that for me, it will not help. Truly speaking we have no rights on this*.

#### Perceptions of being weak, and powerlessness

Some participants revealed that they did not attend school, uneducated and as a result, perceived having low health literacy and so weak to demand their healthcare rights. Health care providers also felt that most patients were medically illiterate to understand their health conditions and healthcare rights.

#### Perceptions of fear of violations of hospital regulations

Some patients are concerned that they might violate the hospital regulations unknowingly if they would request or demand their healthcare rights. This perception limits their motivation to demand their rights in healthcare.

*For example morning times when it is cold, you may sit down on the bed but they would get angry and scold at you, but it should have to be in a polite way like ‘get up please, this is not allowed, and it is only for patients’, This is somewhat problematic.-20-year-old, female patient*

### Suggested strategies to promote patients right in healthcare and medical consultations

Patients and health workers agreed that it is essential to implement strategies that inform and empower patients to demand and use their rights while seeking medical care. The strategies suggested by participants included the following;

#### Educating patients about their healthcare rights and how to use their rights

It was mentioned that the hospitals and health workers have the primary responsibility to teach and create awareness about patient’s rights.

*We should teach patients about their rights. If patients and their caretakers are conscious of their rights, it would be easy for providing care. We should teach them that they have the right to get a bed and take their medication properly and voluntarily.- (KII, Health worker)*.

Education about patient’s rights should be addressed as part of the patient education program and sessions in the hospitals and health care facilities. It was recommended that hospitals should use various educational methods including print media (display on walls) and face-to-face educations for awareness creations. A health worker said,

*Health education should have to be given every morning because the patient should aware of what to do and how to behave in the hospitals while seeking care and in the consultation process*.

Another key informant (health worker) also added on the need to educate patients on the care delivery process in the hospital setups.

*Rather than getting into conflict with workers for what they don’t have information about it they have to be well informed. I would suggest that they have to be informed and know where they should go to the hospital to get certain services. They have to identify when services are available particularly for none emergency services*

Some key informants mentioned that educating patients and caretakers about the different roles, responsibilities of service providers in health facilities, and different professional categories who are engaged in providing services could help to promote patient’s healthcare rights. It is believed that if patients are aware of who is doing what in the caregiving process, it would be easier for them to direct their questions and demands to appropriate health personnel in health facilities.

*In my view*, *many patients do not recognize the difference in job descriptions health professionals have*. *For example*, *there are nurses*. *Their role could be making beds comfortable for the patients and medication administration*. *Doctors can also provide such services*, *but from the job description point of view providing patient care is the duty of the nurses*. *The majority of the patients think that all services are carried out by doctors*. *When some problems happen*, *every complaint goes to doctors*.*-* (KII, Health, worker)

### Educate guards on patient rights and patient handling

*I have an identification card given to enter and exit from the hospital freely. But the rigid guards who are throwing offensive words and do not allow you to enter the hospital though you have entrance ID, saying-‘Moche egegnalehu esky kegebu’–as long as I am alive you will never enter to this hospital. For such rigid people, education should be given because patients may die if not permitted. - 30-year-old, woman*.

## Discussions

In the process of healthcare and medical consultations, the patient has the right and obligations as well [2, 3]. In this study, we assessed patients’ rights during hospital visits from the perspectives of patients, caretakers, and health care providers in Oromia, Ethiopia. Accordingly, the study revealed that patient has several legitimate rights which are organized under three major thematic areas, namely organizational or structural, socio-cultural, and clinical care, with cross-cutting and supportive elements (i.e. education, counseling, engaging, and empowering) embracing several specific rights within each major theme including the right to access to care treatment, diagnosis and investigations, access to drugs and medications. Even though the right-based approach to healthcare would promote the fulfillment and protection of patients’ rights as human beings, including dignity autonomy, equality and respect, participation and interdependence, and fairness in treatment [6–10], patients’ concerned that their rights were neither fulfilled nor protected in practice.

Consistent with the existing patient rights framework and deceleration [6–10, 29], patients’ accorded that they have the fundamental right to adequate and responsive clinical care, expressed as access to prompt care, medical investigations, effective treatment, and follow up care that continually maintained (ensured continuity of care). Indeed, responsiveness is one of the indicators for quality health care system-delivering the right care at the right time [30]. Inconsistent with this expectation, patients were concerned that they did not get prompt care in most of the circumstances, and their right to get prompt care was not fulfilled. This suggests that patients’ right to prompt care should be a priority of focus for health managers in the current setting and it is essential to put in place processes to ensure and safeguard the rights of patients to get adequate and timely care. In this regard, patients’ concerns were mostly related to delayed laboratory investigations and notifications of results, and thus, important to set clear timelines and service delivery standards to eliminate unnecessary delays in the process of care.

Closely linked to clinical care, the right to make informed decisions and informed choices were another focus area for the fulfillment of patients’ rights as consistently agreed-upon research participants. It was frequently reflected that the views and preferences of patients must be heard and taken into account in providing services and making a decision regarding patient care. This suggests that patients want to have participatory and shared decision-making on their matters, including the right to include their families in the decision-making process. Thus, health care providers required to provide information about patients’ diagnosis, prognosis, and the different treatment choices, address patients’ patient preferences, share evidence behind the recommendation of a certain course of treatment, and describe alternative options, ask for patient input and modify the plan, if necessary. This will increase citizen’s confidence, trust, ownership, and could support the right-based approach to healthcare delivery [9, 31]. Taking into consideration these perspectives, patients and health workers made clear that patient has the right to decide on a major aspect of their care including the right to procedure, and medications that avoid unnecessary discomfort. Indeed, in settings where medical literacy is generally low leaving these critical decisions for patients may not possible, rather educating and empowering patients for an informed joint decision could be helpful and should be prioritized by the care providers and managers.

This study also uncovered that patients valued continuity of care and felt that it is their right to be fulfilled, patient follow-up care in terms of scheduled visits, and monitoring of the treatment progress. Follow-up care is essential to ensure continuity of care, better clinical outcomes, and building a long-term relationship with the patients and families, and communities at large. In contrast, patients are concerned that this basic right was not protected and suggested for a stronger system that ensures effective patient follow-up, treatment updates, and adjustments, and an improved feedback system to meet patients’ continuing health care needs even after discharge. It is also important that health care providers should provide clear follow-up plans for patients and families to fulfill patients’ demands. In connection with continuity of care, patients’ and caretakers’ strongly demanded that they had the right to be treated by the same doctor or care providers during their visit(s), especially when admitted for inpatient care, making right be treated by the same doctor, the most frequently demanded and emphasized rights by patients and caretakers Many patients’ right declarations also underscore that the patient has the right to know the practitioner who is primarily responsible for his/her care including the names, professional type or jobs, and identity [2, 3]. Indeed, being treated by the same provider would help to establish and maintain a good therapeutic relationship and also helpful for the doctor to monitor the patient’s health progress and response to treatment effectively and efficiently. In contrast, patients mostly worried that there was a frequent change of their care provider’ which shouldn’t happen in the future for the sake of providing better care for patients. This is typical challenge patients were facing in referral and teaching hospitals where clinical rotations are incorporated in medical education so that the trainees have access to patients to gain hands-on medical experience. Perhaps, the hospitals are required to develop/enforce a clear guideline and system of accountability for the handover of patients between shifts or rotations. Also, educating patients regarding the need for and significance of health professionals’ training and rotation may help to reduce patients’ discomforts.

Referral for higher care and transfer to other facilities based on patient’s need and a request was perceived to be patients’ right. Certainly, poor referral linkage is one of the critical challenges in a less developed health system [24, 32, 33]. Thus, it is essential to strengthening referral networks that can effectively link health facilities, both vertically and horizontally. Indeed, the realization of such rights needs to be made with caution not to put patients’ lives at risk during transportation or travel. It requires insurance of effective referral arrangements between facilities and the patient has to be given complete information about the process and the hospital’s obligations.

Illness (eg. presentation and response) and medical care (eg. communication, relational and interactions, cultural) are largely influenced by socio-cultural domains [34–36]. Interestingly, study participants in the present context implied that patient a has the right to receive socio-culturally competent care which included the right to openly express personal illness trajectory, and communicate in their language according to the culturally appropriate way of communication; the right to be treated with due respect dignity and compassion by the staffs and doctors in the hospitals. There was a common agreement that a patient has the right to openly express their feelings and tell their illness story to their doctors in their language and the right to know their illness in the language they can understand. To fulfill this particular right, health care providers should allow and encourage patients to tell their illness stories and must develop a good relationship and empathy to properly respond to patients’ emotions. Of course, it is recognized more than ever that effective patient care depends on effective interpersonal communication that would enable the doctor to gather detailed patients’ histories and help a patient to understand his/her illness for better cooperation and self-care practices. In addition, it could contribute to patient empowerment and enablement [13]. However, no health workers tell patients’ illnesses and it should be improved for two reasons; respecting the right of patients and ensuring patient engagement in their care. Earlier studies also reported similar phenomenon [14–16, 18, 25, 26, 37].

Even though the right to respectful care was one of the rights patients valued most, there was a widespread concern that health staffs especially non-technical staff such as security/guards, patient runners, and cleaners often disrespect, verbally abuse, and humiliate patients. Disrespect, lack of empathy mistreatment, and poor patient handling are among the common challenge to providing respectful care to the population in less developed health care systems [15]. This requires the need to devise a policy and package towards the right based approach through compassionate, empathetic, respectful, and caring health professionals. Moreover, establishing a system of accountability for disrespecting and disgracing patients’ training, and encouraging health professionals to be respectful and compassionate to their patients and clients is crucial to promote respect and dignity as core patients’ right healthcare delivery process. The development of caring, respectful, and compassionate health workers may require multiple approaches from reforming the recruitment of students for health science training to improving the curriculum of the various disciplines towards socio-cultural sensitive care, and effective on job capacity building interventions for the health professionals.

The public health care system is the universal, irreligious, non-sectarianism and ultimate purpose to serve the public equally without any discrimination and prejudice [8–10, 38]. In line with this principle, a patient has the right to get health service in the language they understand, and hence, it is vital to establish a clear and transparent system that would enable patient’s rights to be fulfilled and patients are served in their language without any discriminations. In contrast, patients were highly worried that they were discriminated against (partial treatment) because of their background, especially due to language barriers. It appeared that hospitals staffs especially supportive staffs tend to favor people who speak a certain language and culture over other ones, especially Afan Oromo speakers complained that they were disregarded, the level of care given to them was compromised and they were humiliated because of the language they were speaking. Those patients explicitly stated that the patients have the right to be served by their language and hence, alleviating language barriers and all forms of discrimination against patients must be urgent action and priority in the health care system, to respect patients healthcare rights. Given that Ethiopia is a multi-national country with people from diverse backgrounds and languages, it is crucial to develop a clear language policy in the health care delivery system especially in secondary and referral hospitals where patients are expected to be from different backgrounds. Failure to avoid language barriers in healthcare consultation is a volition of fundamental human rights, essentially puts patients’ lives at risk, and generally against the core value of the healthcare system [8, 31, 38]. As stipulated in the Ethiopian constitution, the right to health is a constitutional right and must be respected by the healthcare system [22]. Thus, the regional and federal governments should devise viable laws and short and long-term strategies to address language barriers in healthcare to protect the right of citizens to equitable and quality healthcare. It also imperative that health managers should implement strategies such as language training to staff, offering language assistance services including interpreter services, recruiting multi-lingual staffs at some critical service delivery points, and re-orient its service delivery scheme to respect diversity, equality culture, and fair treatment of patients visiting the hospitals.

The right to the organizational and structural aspects of care was another crucial dimension of patients’ perceived right in the present context. In this emergent theme, one of the rights patients stressed was problems related to the right to access their medical cards and documentation. In this regard, despite participants widely recognized that it is a patient’s right to access medical records and all information contained within it within a reasonable timeframe, the patient often gets this basic right was not protected. Actually, in resource-poor settings establishing a well-organized patient medical record and filing system was often lacking which makes searching and retrieving patients’ medical cards might be tiresome and difficult, leading to unnecessary discomfort, and violations of their right to timely access to medical care.

The right to access and the use of supportive facilities and resources available in the healthcare setting was a top concern to patients. In this regard, patients were seriously discussed about the right to access to clean water for drinking and other purposes, and the right to access to toilet and sanitation facilities while visiting and staying in hospitals. Nevertheless, patients’ right to access such important facilities appeared restricted or limited in many cases. For example, many patients complained that the toilet was not opening especially at night, and they also did not familiar with the toilet design and sanitation facility setups (e.g. lack of responsiveness and adaptability of facilities). In conjunction with this, patients recognized that not only the right to use facilities but also a patient has the right to get information, educations, and instructions on the proper use of these facilities and the right to know what facility rules and regulations applicable to his/her conduct as a patient. In this study, however, there was evidence suggesting patients’ right to access to information, education, guidance, and assistance was non-existent.

As hospital facilities are meant to serve patients, it is a valid question that the hospital’s managers are needed to respond to patient inquiry through education, guidance, and creating a conducive and supportive hospital social and physical environment for patients. Moreover, patients had serious concerns over the right to have caretakers while visiting hospitals-they reflected that the hospitals mostly forbid families and caretakers from visiting sick persons while in the hospital. instead of disallowing caretakers and visitors, hospitals are required to establish clear guidance regarding caretakers and their engagement in the patient care process. On the other hand, it was perceived that caretakers would have the right to get a suitable resting place, and hospitals were criticized for not having a separate resting place or arrangement for caretakers and families especially at night.

Remarkably, participants (especially patients) were well aware of the right they have as a patient to be treated and cared in a safe environment that does not expose them to health hazards and infections. This will have significant implications for hospitals’ managers and the health system at large for strict implementation of infection prevention and control measures to ensure patients’ and caretakers’ safety. As part of ensuring this aspect of rights, it is important to teach patients and caretakers on risk reduction behaviors and adaption of appropriate safety and self-protective measures while visiting hospitals. Closely related to the right to infection prevention, patients claimed the right to get some self-protective tools and supplies such as mosquito nets to prevent themselves from a mosquito bite and of course malaria. This is an important notification for the hospital management for further evaluations and actions to ensure no risk happens to patients. The study also uncovered that access to safe and adequate food services or supplies of their preference, especially when recommended by their doctors or dietetics together with diet education and counseling service was patients’ rights. This calls for the need to strengthen patient education programs and counseling services to satisfy the need and rights of patients.

The study also identified key barriers to implementing patient’s rights which are largely related to an interplay of factors such as lack of patient empowerment (eg. patients’ sense of dependency, feeling of powerlessness, fear of consequence such as behavioral insult, rejections, being fired, or denied services if they speak out to demand their rights), lack of patient engagement in the process of care, and lack of patient education and counseling. As a crosscutting and supporting environment for the realizations of patients’ right during the health visits, these concepts (education, empowerment, engagement and counsel) emerged as the codes were clustered together at higher level and altogether it was recognized patient’s fear to ask for one’s own right (needs empowerment), lack of awareness on how demand and ask their (needs education); fear of consequences (needs counseling), and some rights such as lack of involvement in decision making because of lack of sense of dependency (needs engagement). On the other hand, health care provider and system factors such as negligence, lack of awareness and recognition of patient rights, perceptions of low literacy patients, mistreat and disrespect also require establishing system of accountability and empowerment of health workers. Perhaps, lack of a system or policy framework that guides providers towards respecting and fulfilling patient’s rights contributed to the lack of respect for patients ‘rights. The study identified valid views and strategies suggested by participants that may help to overcome these barriers and ensure patient’s rights are protected during medical consultations. Such strategies included informing, educating, and empowering patients about their rights and how to safely exercise their rights; educating and re-orienting health staffs perceptions and awareness on patients’ rights and the need to obey patients in all aspects, and developing and implementing patient rights legislative framework and policy that guides health care delivery process.

### Strengths and limitations of the study

The study employed a qualitative approach with a relatively large sample and diverse populations (patients in group discussions, caretakers, in-depth interviews, and diverse groups of health care providers) which enabled the researchers to explore deep beliefs, experiences, and perceptions of patients and health staff regarding patient’s rights in the healthcare. The study yield depth insight into the study objectives and we believe that it ensured idea representation on the phenomenon which is one of the quality indicators in qualitative researches. To the best of our review and knowledge, this study is the first of its kind in Ethiopia in exploring patient’s rights in health. However, the study was conducted involving four hospitals and may not fully reflect the perceptions and experience prevailing in Ethiopia, in general and further studies are required on this developing subject. In addition, we did not assess the source of the patients’ knowledge concerning their rights, rather the study was focused on exploring and constructing their perceptions and experience. Moreover, the findings did not address rights specific to certain demographic characteristics such as patients’ age, gender, and literacy, among others.

## Conclusions

Patients’ rights to access to healthcare are a fundamental human right, but often depending upon prevailing health system arrangements and socio-cultural factors. This study documented patients’, caretakers’, and health care providers’ perceptions of patients’ rights to healthcare during hospital visits for medical consultations and clinical services. The perceived patients’ rights to healthcare encompassed three main aspects: the rights to appropriate clinical care, the right to access care that fits the socio-cultural and humane contexts, and the right to access services in an organizationally and structurally coordinated and legislatively supported manner. Specifically, a wide range of patients’ rights that were established included the right to access clinical care, treatment, and services with prompt and timely diagnosis, based on the patients’ informed decisions, through respect to human dignity, and in their language without any discrimination. It was reflected that patient has fundamental rights to access to adequate and prompt care, access to right medications, right to be treated by the same provider and right to share their illness history and right to know their illness, right to engage in their care for informed decisions and choices with the right to get follow-up and updates on the health conditions. In addition, patients also perceived that they have entitled to claim rights such as the right to access to the use of resources and facilities available in hospitals without restrictions, the right to receive education and information, the right to be protected from risks and hazards that may be encountered while seeking care in hospitals. Despite well-recognized rights patients’ may have during health facility visits; there was a general agreement that these rights were mostly unmet in practice. Several factors that were profoundly related to patients (fear of consequence, perceptions of dependency), health care providers (negligence, lack of awareness and recognitions), and systems (lack of policy guiding framework) contributed to lack of respect and violations of patients’ rights. Therefore, this finding has significant policy implications to inform policy and prioritize implementation of patients’ rights charter in Ethiopia based on a rights-based approach to healthcare and clinical services-the health systems are required to develop a clear patient rights framework for a rights-based approach to patient services; inform, educate and empower patients to know their rights and demand during medical consultations. Moreover, it is essential to train and re-orient health care providers to uphold patient’s rights along with putting in place a legislative framework to reinforce the realization of these rights. Likewise, patients’ recognitions and knowledge concerning their rights during health facility visits could help the health system to inform the development of tailored educational components and can facilitate the implementation of legislative measures at facility levels. Finally, it is important to conduct further studies including quantifications and statistical validations of the perceived rights explored in the present study.

## Supporting information

S1 Appendix Curriculum Vitae of the data coder (ZB). (PDF)

S2 Appendix Curriculum Vitae of the data coder (YK). (PDF)

S3 Appendix List of emergent codes and their members. (PDF)

S4 Appendix Information on the consolidated criteria for reporting qualitative research (COREQ). (PDF)
