# Supplementary material for: Patients’ healthcare, education, engagement, and empowerment rights’ framework: Patients’, caretakers’ and health care workers’ perspectives from Oromia, Ethiopia
Source: PLoS One. 2021 Aug 12;16(8):e0255390. doi: 10.1371/journal.pone.0255390 (PMC8360507; doi:10.1371/journal.pone.0255390)
Supplement: S1 Appendix — (PDF) [file pone.0255390.s001.pdf]

Curriculum Vitae

Personal information

First name(s) / Surname(s)	**Zewdie Birhanu Koricha (P.h.D)**		
Address(es)	Jimma town, Oromia region , Ethiopia		
Telephone(s)		+251917025852<br>+251471114439	Mobile<br>Office
E-mail	zbkoricha@yahoo.com, or Zewdie.birhanu@ju.edu.et		
P.O. Box	378, Jimma, Ethiopia		
Nationality	Ethiopian		
Date of birth	12/09/ 1982		
Gender	Male		

Work experience and position

Position and ranks	**July 2006- Sep 2007**	Graduate assistant
	**July 2009- June 2012**	Lecturer of Health Education and Promotion
	**June 2012-June 2016**	Assistant professor of health education and promotion
	**March 2016 onwards**	Associate professor at department of Health, Behaviour and Society, Faculty of Public Health, Jimma University

**Current position held/rank**	**Associate professor and Head, Department of Health, Behaviour and Society, Institute of Health, Jimma University**
Main activities and responsibilities	Teach undergraduate, distance learners and graduate students various courses (healthy behaviour, health promotion, health communication, health education, health promotion ethics, qualitative research, strategic communication, statistical analysis in behaviour ral studies)<br>Participate in field works, student supervisions during attachments<br>Advised several undergraduate and graduate students during their thesis work<br>Participated in consultancy services<br>Undertaken/participated in research projects<br>Participated in community services<br>Offer/facilitated trainings<br>Prepared distance learning materials<br>Member of ethical review board of College of Public Health and Medical Sciences, Jimma University
Positions	Head, department of Health, behaviour and Society (July 2017-August 2019)<br>Chief academic and research directors (August 2019-March 2020)<br>Served as Ethics review committee member (2010-2013)<br>Served as member of COVID-19 advisor council for Oromia regional state (March –June 2020)
Name and address of employer	Kora Tushene<br>Jimma town, Oromia region, Ethiopia<br>Phone: +251911241916<br>e-mail: kora.tushune@ju.edu.et
Name and address of organization	Jimma University, Oromia, Ethiopia
Type of business or sector	Public University

Education

Degree awarded	GPA and class	**Dates**	**Principal subjects covered**
**PhD** in infectious and tropical diseases	Excellent (research grade)	June 2017	Public Health, infectious disease, entomology, epidemiology and biostatistics, research policy and ethics, zoonotic diseases, molecular techniques, bio-informatics, malaria epidemiology and preventive strategies
**MPH** degree in Health Education and Promotion	3.83 (Great distinction)/ first class	Sep 2007-June 2009	Education promotion, health communication, healthy behaviours, behavioural models and theories, Biostatistics, epidemiology, reproductive health, health service management, research methodologies
**BSc** degree in Health Education and Promotion	3.85 (great distinction) first class	Sep 2003-June 2006	Health Education, Health promotion, Public health courses including epidemiology and Biostatics

Short term trainings

Title of qualification/certificate awarded	Dates	Principal subjects covered	Name and type of organisation providing training
1. Certificate of facilitator for Social marketing for Health and Development	21st -25th March 2011	Facilitated the training on social marketing planning process for development workers	AfricomNet in collaboration with Jimma University
2. Certificate of successful completion of trainings on communication material development	April 4-9, 2011	Communication material development, design, production, pretesting, P-process	Johns Hopkins University in collaboration with AIDS resource centre of Ethiopia
3. Certificate of successful completion of training on communication for Change 'C-CHANGE'	December 12-20th , 2011	Strategic communication planning process, behavioural models and theories	African Network for strategic communication (AfriCOmNet)

4.	Certificate of successful completion of training on qualitative research methodology with MAXQDA software application	December 26-29, 2011	Qualitative research methodology: Designs, methods, planning and conducting qualitative research, data analysis using MAXQDA	Jimma University in collaboration with VILR-IUC (Belgium University)
5.	Certificate of successful completion of training for institutional review ethics committee and researchers	6-10 February 2010	Health research Human protection, GCP and SOP	Ethiopian Bioethics Initiative (ETBIN) in collaboration SIDCER EDCTP, African Bioethics Imitative
6.	Certificate of attendance on "Systematic review"	22nd November -3rd December 2010	Introduction to evidence based health care and the systematic review of the evidence<br>The appraisal, extraction and pooling of quantitative data from excremental, non-experimental, diagnostic and prognostic studies<br>The appraisal, extraction and pooling of qualitative data from qualitative studies, narratives, and text from opinion	The JOANNA BRIGGS institute (Australia) in collaboration with Jimma University
7.	Certificate of course participation and successful completion of training on scientific paper writing	6-9 Oct 2009	Scientific paper writing skill, research methodologies, publications of scientific paper	Joint Cooperation of Jimma University and VILR-IUC (Belgium University) program
8.	Certificate of successful completion of training on Module writing in higher	November 2-5, 2009	Distance module writing principles, methods, pedagogical and subject matter considerations, steps	Jimma University
9.	Certificate of successful completion of training on community mobilization	September 22-26, 29006	Community mobilization process and action cycle, planning, implementation, monitoring and management, evaluation of community mobilization process	Jimma Univeristy in Collaboration with AfriComNet
10.	publication management	3-4 Oct 2013	Publication management, role of reviewers, editors, authors, ethical issues in publications	African journal Partnership Project
11.	Social Behavioral Change Communication for Malaria workshop	17-21 Sep 2013	Behavior Change Communication (BCC) for malaria, theories/models, best experiences, monitoring and evaluation of malaria activities	PMI, USAID
12.	Qualitative data analysis and management using ATLAS ti.7	April 29-31, 2014	Preparing data for analysis, coding, code management, creating code families, analysis, network management, creating report, building theory	Resilient African Network
13.	Scientific writing	July 2015	Writing scientific papers, reference manager tools	Author aid
14.	Implementation research	Feb 2018		
15.	Grant writing	March 13-15, 2019, JU	Grant writing, academic wring	Texas Tech University professors
16.	Comprehensive Systematic Review Training Program:	3 February 2020 – 7 February 2020	Module 2: Conducting Systematic Reviews of Quantitative Evidence<br>(2-day workshop)	Joanna Briggs Institute

Personal skills and competences

Mother tongue(s)	Afan Oromo, Amharic				
Other language(s)	English				
Self-assessment	**Understanding**		**Speaking**		**Writing**
European level (*)	Listening	Reading	Spoken interaction	Spoken production	
**Language- English**	B2 (independent user)	C1 (proficient user)	B2 (independent user)	B2 (independent user)	C2 (proficient user)

Computer skills and competences: Advanced skill on Microsoft word and Microsoft PowerPoint, Moderate skill on Microsoft excel and Microsoft publishers

Advanced skill on SPSS statistical data analysis and ATLAS-ti qualitative data analysis software; basic skill on STATA, JBI systematic review software (Cochrane) and Qualitative MAXQDA software

Peer reviewed publications

1. **Zewdie Birhanu,** Tsion Assefa, Mirkuzie Woldie, Sudhakar Morankar. Determinants of satisfaction with health care provider interactions at health centers in Central Ethiopia: a cross sectional study. BMC Health Services Research 2010, 10:78
2. **Zewdie B. et al**. Assessment of production and distribution of printed Information Education Communication (IEC) materials in Ethiopia and utilization in the case of jimma zone, Oromiya national regional state: a cross sectional study; Ethiop J Health Sci. Vol. 1, No. 1 August, 2010
3. **Birhanu Z,** Woldie M, Assefa T, Morankar S.Determinants of patient enablement at primary health care centres in central Ethiopia: a cross-sectional study. Afr J Prm Health Care Fam Med. 2011;3(1)
4. **Zewdie Birhanu**, Mirkuzie Woldie, Tsion Assefa, Sudhakar Morankar. Determinants of perceived empathy among patient attended primary health care centers, central Ethiopia. International Journal for Quality in Health Care 2012; pp.1–8
5. Amare Deribew , Fessehaye Alemseged, **Zewdie Birhanu** et al. Effect of training on use of Long Lasting Insecticide treated bed net on the burden of malaria among vulnerable groups, south west Ethiopia: Baseline result of a cluster randomized trial. Malaria Journals 2010,9:121
6. Deribew A, Alemseged F,Tessema F, Sena L, **Birhanu Z**,etal.(2010). Malaria and Under Nutrition:A Community BasedStudy Among Under-Five ChildrenatRiskofMalaria,South-WestEthiopia.PLoSONE5(5).
7. Amare Deribew, **Zewdie Birhanu**, Lelisa Sena et al. The effect of household heads training about the use of treated bed nets on the burden of malaria and anaemia in under-five children: a cluster randomized trial in Ethiopia, Malaria Journal 2012, 11:8.
8. Amare Deribew, **Zewdie Birhanu**, Lelisa Sena et al. The effect of household heads training on long lasting insecticide-treated bed nets utilization: a cluster randomized controlled trial in Ethiopia. MalariaJournal 2012, 11:99
9. Endalew Hailu, Tefera Belachew, Wudineh H/Mariam, **Zewdie Birhanu**. Self-care practice and glycemic control among adults with diabetes at Jimma University Specialized Hospital, South West Ethiopia: A cross sectional study. Afr J Prm Health Care Fam Med.2012;4(1).
10. DejeneTilahun, **Zewdie Birhanu**: Effect of community based behavioural change communication intervention to improve neonatal mortality in developing countries: A Systematic Review. JBI Library of Systematic Reviews; 2011;9(40):1650-1679
11. Yohannes, Kebede Lemu, **Zewdie Birhanu Koricha**, Lakew Abebe Gebretsadik , Ameyu Godesso Roro. Predictors of refusal of provider initiated HIV testing among clients visiting adult outpatient departments in Jimma town, Oromia Region, Ethiopia: unmatched case control study. HIV/AIDS - Research and Palliative Care 2012:4 103–115
12. **Zewdie Birhanu** Alemseged Abdissa, Tefera Belachew, Amare Deribew, Hailemariam Segni, Vivien Tsu, Kim Mulholland, Fiona M Russell. Health seeking behavior for cervical cancer in Ethiopia: a qualitative study. International Journal for Equity in Health 2012, 11:83
13. **Zewdie Birhanu**, Ameyu Godesso, Yohannes Kebede and Mulusew Gerbaba. Mothers' experiences and satisfactions with health extension program in jimma zone, Ethiopia: a cross sectional study**.** *BMC Health Services Research* 2013, **13**:74
14. Bezawit Temesgen, **Zewdie Birhanu,** Tigist Astale, and Tariku Dejene. Predictors of intended infant feeding options among HIV positive pregnant women in Addis Ababa: The perspective of theory of planned behaviour. J. AIDS HIV Res 2013, 5(7), pp. 260-268.
15. Bedada T., Belachew T., Hailu E., **Birhanu Z**. Glycogenic Control and Associated Factors among Diabetic Patients Visiting Adama Specialized Hospital, Oromiya, Ethiopia: A Facility Based Cross Sectional Study. *Afr J Health Sci. 2013; 26:302-313*
16. Doyore F, **Birhanu Z,** Kebede Y, Dejene T,Jara D (2013) Are People Controlling the Danger or Fear for Condom Use as HIV/AIDS Preventive Message? An Evaluative Type of Study Based on Extended Parallel Process Model. J AIDS Clin Res 4: 264.
17. Addisu Y, **Birhanu Z,** Tilahun D, Assefa T. Predictors of treatment seeking intention among people with cough in East Wollega, Ethiopia based on the theory of planned behavior: a community based cross-sectional study. Ethiop J Health Sci. 2014 Apr;24(2):131–8.
18. Nagasa Dida, **Zewdie Birhanu,** Mulusew Gerbaba, Dejen Tilahun, Sudhakar Morankar. Modeling the probability of giving birth at health institutions among pregnant women attending antenatal care in West Shewa Zone, Oromia, Ethiopia: a cross sectional study. *African Health Sciences 2014; 14(2):288-298*
19. **Birhanu et al.** Does Life Satisfaction Correlate with Risky Behaviors? Finding from Ethiopian Higher Education Students. Global Journal of Research and Review; GJRR[1][1][2], 2014
20. Gebremeskel Mirutse, Girmatsion Fisseha, Lakew Abebe, **Zewdie Birhanu**, Mussie Alemayehu. Intention to Donate Blood among the Eligible Population in Mekelle City, Northern Ethiopia: Using the Theory of Planned Behavior, American Journal of Health Research. Vol. 2, No. 4, 2014, pp. 158-163.
21. Yamrot Debela, **Zewdie Birhanu,** Yohannes Kebede, Malaria Related Knowledge and Child to Parent Communication Regarding

Prevention and Control of Malaria among Primary School Students in Jimma Zone, South West Ethiopia, American Journal of Health Research. Vol. 2, No. 5, 2014, pp. 284-290. doi: 10.11648/j.ajhr.20140205.20

22. Dereje Geleta, **Zewdie Birhanu,** Michelle Kaufman, Bezawit Temesgen. Gender Norms and Family Planning Decision-Making Among Married Men and Women, Rural Ethiopia: A Qualitative Study. Science Journal of Public Health 2015; 3(2): 242-250
23. **Birhanu Z**, Abebe L, Sudhakar M, Dissanayake G, Yihdego Y, Alemayehu G, et al. Access to and use gaps of insecticide-treated nets among communities in Jimma Zone, southwestern Ethiopia: baseline results from malaria education interventions. BMC Public Health. 2015 Dec 29;15(1):1.
24. Martin S, Omotayo M, Chapleau G, Stoltzfus R, **Birhanu Z**, Ortolano S, et al. Cross-Country Comparison of the Acceptability of a Social Support Behavior Change Strategy to Improve Adherence to Antenatal Calcium and Iron-Folic Acid Supplementation. FASEB J. 2016 Apr 1;30(1 Supplement):422.7-422.7.
25. **Birhanu Z,** Abebe L, Sudhakar M, Dissanayake G, Yihdego Y, Alemayehu G, et al. Malaria Related Perceptions, Care Seeking after Onset of Fever and Anti-Malarial Drug Use in Malaria Endemic Settings of Southwest Ethiopia, Plose One (in press, acceptance date: July 15, 2016
26. Martin SL, Omotayo MO, Chapleau GM, Stoltzfus RJ, **Birhanu Z**, Ortolano SE, et al. Adherence partners are an acceptable behaviour change strategy to support calcium and iron-folic acid supplementation among pregnant women in Ethiopia and Kenya. Maternal & Child Nutrition. 2016 Jan 1;n/a-n/a.
27. Neno Nikus, Mamusha Aman, **Zewdie Birhanu**. HIV stigma and associated factors among antiretroviral treatment clients in Jimma town, Southwest Ethiopia. HIV/AIDS - Research and Palliative Care 2016:8 1–11
28. **Birhanu,** Z., Chapleau, G. M., Ortolano, S. E., Mamo, G., Martin, S. L., and Dickin, K. L., Ethiopian women's perspectives on antenatal care an iron- folic acid supplementation: Insights for translating global antenatal calcium guidelines into practice,Matern Child Nutr, 2016. doi: 10.1111/mcn.1242410 .
29. Martin SL, **Birhanu Z,** Omotayo MO, Kebede Y, Pelto GH, Stoltzfus RJ, et al. "I Can't Answer What You're Asking Me. Let Me Go, Please." Field Methods [Internet]. 2017 May 24 [cited 2017 May 25]; Available from: http://journals.sagepub.com/eprint/HEqFMQKcbhn78M63vFPn/full
30. **Birhanu, Z.;** Ambelu, A.; Berhanu, N.; Tesfaye, A.; Woldemichael, K. Understanding Resilience Dimensions and Adaptive Strategies to the Impact of Recurrent Droughts in Borana Zone, Oromia Region, Ethiopia: A Grounded Theory Approach. *Int. J. Environ. Res. Public Health* **2017**, *14*, 118.
31. Hadush Z, **Birhanu Z**, Chaka M, Gebreyesus H. Foods tabooed for pregnant women in Abala district of Afar region, Ethiopia: an inductive qualitative study. BMC Nutrition. 2017;3:40.
32. **Birhanu Z,** Yihdego YY, Emana D, Feyissa D, Kenate S, Kebede E, et al. Relationship between exposure to malaria and haemoglobin level of children 2–9 years old in low malaria transmission settings. Acta Tropica. 2017 Sep;173:1–10.
33. Yohannes Ababu, Fiona Braka, Aschalew Teka, Kinde Getachew, Tefera Tadesse, Yohannes Michael, **Zewdie Birhanu**, Peter Nsubuga, Tersit Assefa, Kathleen Gallagher. Behavioral determinants of immunization service utilization in Ethiopia: a cross-sectional community-based survey. Pan African Medical Journal [Internet]. 2017 Sep 6 [cited 2017 Jun 10];ARTVOL(ARTISSUE). Available from: http://www.panafrican-med-journal.com/content/series/27/2/2/full/#.WTunjjeqrIU
34. Ambelu A, **Birhanu Z**, Tesfaye A, Berhanu N, Muhumuza C, Kassahun W, et al. Intervention pathways towards improving the resilience of pastoralists: A study from Borana communities, southern Ethiopia. Weather and Climate Extremes [Internet]. [cited 2017 Jun 20]; Available from: http://www.sciencedirect.com/science/article/pii/S221209471730004X
35. Gezahegn T, **Birhanu Z**, Aman M, Dessalegn M, Abera A, Nyagero J. Peer communication on sex and sexual health among youths: a case of Debre Berhan university, Ethiopia. Pan Afr Med J [Internet]. 2016 Nov 26 [cited 2017 Jul 11];25(Suppl 2). Available from: http://www.ncbi.nlm.nih.gov/pmc/articles/PMC5390071/
36. Beharu M, Bekele I, Birhanu Z, Yimam I. Cultural Sensitiveness in Health Care Delivery of Jimma University Specialized and Teaching Hospital, South West Ethiopia, 2016. Quality in Primary Care [Internet]. 2017 Jun 1 [cited 2017 Jul 14];25(3). Available from: http://primarycare.imedpub.com/abstract/cultural-sensitiveness-in-health-care-deliveryrnof-jimma-university-specialized-and-teachingrnhospital-south-west-ethiopia-2016-19669.html
37. **Zewdie Birhanu**, Yemane Ye-ebiyo Yihdego and Delenasaw Yewhalaw. Caretakers' understanding of malaria, use of insecticide treated net and care seeking-behavior for febrile illness of their children in Ethiopia. BMC Infectious Diseases (2017) 17:629.
38. **Zewdie Birhanu1**, Kora Tushune, Mulusew G. Jeben. Sexual and Reproductive Health Services Use, Perceptions, and Barriers among Young People in Southwest Oromia, Ethiopia. Sci.2017;28(1):37

39. Tefera Tadesse[1,&], Kinde Getachew[1], Tersit Assefa, Yohannes Ababu, Tesfaye Simireta[2], **Zewdie Birhanu[1]**, Yohannes Hahile Michael[1]*Factors and misperceptions of routine childhood immunization service uptake in Ethiopia: findings from a nationwide qualitative study. The Pan African Medical Journal. 2017;28:290.* doi:10.11604/pamj.2017.28.290.14133
40. **Zewdie Birhanu**, Yemane Ye-ebiyo Yihdego and Delenasaw Yewhalaw. *Quantifying malaria endemicity in Ethiopia through combined application of classical methods and enzyme-linked immunosorbent assay: an initial step for countries with low transmission initiating elimination programme*. Malaria Journal 2018**17**:152
41. Bereket Epheson, **Zewdie Birhanu**, Dessalegn Tamiru, and Garumma Tolu Feyissa. Complementary feeding practices and associated factors in Damot Weydie District, Welayta zone, South Ethiopia. BMC Public Health (2018) 18:419
42. Feleke Doyore Agide Gholamreza Garmaroudi Roya Sadeghi Elham Shakibazadeh Mehdi Yaseri Zewdie Birhanu Koricha Bereket Molla Tigabu: A systematic review of the effectiveness of health education interventions to increase cervical cancer screening uptake. *European Journal of Public Health*, Volume 28, Issue 6, 1 December 2018,
43. Animut M, Mamo A, Abebe L, Berhe MA, Asfaw S, Birhanu Z. "The sun keeps rising but darkness surrounds us": a qualitative exploration of the lived experiences of women with obstetric fistula in Ethiopia. BMC Women's Health [Internet]. 2019 Feb 26 [cited 2019 Mar 1];19(1):37. Available from: https://doi.org/10.1186/s12905-019-0732-3
44. Teshome Shiferaw, Getachew Kiros, Zewdie Birhanu, Hailay Gebreyesus, Tesfay Berhe, Mebrahtu Teweldemedhin. Fertility desire and associated factors among women on the reproductive age group of Antiretroviral treatment users in Jimma Town, South West Ethiopia. BMC Res Notes (2019) 12:158. https://bmcresnotes.biomedcentral.com/articles/10.1186/s13104-019-4190-7
45. Befkadu Bekele. Zewdie Birhanu. **Yohannes Kebede**. Megistu M. Koyira**.** ***Intention to HIV Testing Among Pregnant Women, Areka Town, Wolaita Zone, Southern Ethiopia: A Community Based Cross-Sectional Study.*** Developing Country Studies. Vol.6, No.4, pp: 70-81, 2016 ISSN 2224-607X (Paper) ISSN 2225-0565 (Online). www.iiste.org.
46. ***Sabit Ababor, Zewdie Birhanu, Atkure Defar, Kasahun Amenu, Amanuel, Dibaba, Desalegn Araraso, Yosef Gebreyohanes, Mamuye Hadis. Sociocultural*** Beliefs and Practices Influencing Institutional Delivery Service Utilization in Three Communities of Ethiopia: A Qualitative Study. Ethiop J Health Sci. 2019;29(3):343
47. Hailay Gebreyesus, Abebe Mamo, Mebrahtu Teweldemedhin3, Berihu Gidey1, Znabu Hdush4 and Zewdie Birhanu. Experiences of homeless women on maternity health service utilization and associated challenge in Aksum town, Northern Ethiopia BMC Health Services Research (2019) 19:359
48. Feleke Doyore Agide, Gholamreza Garmaroudi, Roya Sadeghi, Elham Shakibazadeh, Mehdi Yaseri, Zewdie Birhanu Koricha, Tadesse Bekele Tefese. Application of Kingdon and Hall Models to Review Environmental Sanitation and Health Promotion Policy in Ethiopia: A Professional Perspective as a Review. Ethiop J Health Sci. 2018;29(2):277. doi:http://dx.doi.org/10.4314/ ejhs.v29i2.15
49. Abebe Mamo, 1 Sudhakar Morankar,1 Shifera Asfaw,1 Nicole Bergen,2 Manisha A. Kulkarni,3 Lakew Abebe,1 Ronald Labonté,3 **Zewdie Birhanu**,1 and Muluemebet Abera4. How do community health actors explain their roles? Exploring the roles of community health actors in promoting maternal health services in rural Ethiopia. BMC Health Serv Res [Internet]. 2019 Oct 21 [cited 2020 Feb 9];19. Available from: https://www.ncbi.nlm.nih.gov/pmc/articles/PMC6805355/
50. Desalegn Dabaro, **Zewdie Birhanu3** and Delenasaw Yewhalaw: Analysis of trends of malaria from 2010 to 2017 in Boricha District, Southern Ethiopia. Malar J (2020). 19:88, https://doi.org/10.1186/s12936-020-03169-w
51. Kebede Y, Yitayih Y, **Birhanu Z,** Mekonen S, Ambelu A. Knowledge, perceptions and preventive practices towards COVID-19 early in the outbreak among Jimma university medical center visitors, Southwest Ethiopia. PLOS ONE. 2020 May 21;15(5):e0233744.
52. Agide FD, Garmaroudi G, Sadeghi R, Shakibazadeh E, Yaseri M, Koricha ZB. How do reproductive age women perceive breast cancer screening in Ethiopia? A qualitative study. African Health Sciences. 2019;19(4):3009–17.
53. Kebede Y, Abebe L, Alemayehu G, Sudhakar M, **Birhanu Z**. School-based social and behavior change communication (SBCC) advances community exposure to malaria messages, acceptance, and preventive practices in Ethiopia: A pre-posttest study. PLOS ONE. 2020 Jun 25;15(6):e0235189.
54. Firanbon Teshome , Yohannes Kebede, Fira Abamecha, Zewdie Birhanu. What women know before Getting Pregnant? Knowledge of preconception care and associated factors among pregnant women in Mana District, Southwest Ethiopia: a community-based cross-sectional study. BMJ Open 2020;10:e035937. doi:10.1136/ bmjopen-2019-035937

55. Gina C. Klemm, **Zewdie Birhanu**, Stephanie E. Ortolano, Yohannes Kebede, Stephanie L. Martin, Girma Mamo, Katherine L. Dickin. Integrating Calcium Into Antenatal Iron-Folic Acid Supplementation in Ethiopia: Women's Experiences, Perceptions of Acceptability, and Strategies to Support Calcium Supplement Adherence. Global Health: Science and Practice 2020 | Volume 8 | Number 3
56. Firanbon Teshome*, Yohannes Kebede, Fira Abamecha and **Zewdie Birhanu**. Why do women not prepare for pregnancy? Exploring women's and health care providers' views on barriers to uptake of preconception care in Mana District, Southwest Ethiopia: a qualitative study. BMC Pregnancy and Childbirth (2020) 20:504 https://doi.org/10.1186/s12884-020-03208-z
57. Yohannes Kebede1* , Lakew Abebe1, Guda Alemayehu2, Morankar Sudhakar1 and **Zewdie Birhanu**. Effectiveness of peer‑learning assisted primary school students educating the rural community on insecticide‑ treated nets utilization in Jimma zone Ethiopia. Malar J (2020) 19:331. https://doi.org/10.1186/s12936-020-03401-7
58. Kebede Y, Alemayehu G, Abebe L, Sudhakar M, **Birhanu Z.** Messenger students' engagement scale: Community perspectives on school-based malaria education in Ethiopia. Health Soc Care Community. 2020;00:1–10. https://doi.org/10.1111/hsc.13193
59. Badassa Wolteji Chala,‡ Zewdie **Birhanu,**† Leta Sera,§ Jemal Abafita* Does Maternal Social Capital Have a Health Payoff? Evidence from Jimma Zone, Ethiopia. African Journal of Economic Review, Volume VIII, Issue III, November 2020
60. Tesfaye Y, Agenagnew L, Terefe Tucho G, Anand S, **Birhanu Z**, Ahmed G, et al. Attitude and help-seeking behavior of the community towards mental health problems. (2020). PLoS ONE 15(11): e0242160. https://doi.org/10.1371/ journal.pone.0242160
61. Kebede Y, **Birhanu Z**, Fufa D, Yitayih Y, Abafita J, Belay A, et al. (2020) Myths, beliefs, and perceptions about COVID-19 in Ethiopia: A need to address information gaps and enable combating efforts. PLoS ONE 15(11): e0243024. https://doi. org/10.1371/journal.pone.0243024
62. Mulugeta Dile Worke1* , **Zewdie Birhanu Koricha2** and Gurmesa Tura Debelew. Prevalence of sexual violence in Ethiopian workplaces: systematic review and meta‑ analysisWorke *et al. Reprod Health (2020) 17:195.* https://doi.org/10.1186/s12978-020-01050-2
63. Firanbon Teshome Gonfa*, Yohannes Kebede Lemu and **Zewdie Birhanu Koricha**, Predictors of Women's awareness ofcommon non-communicable diseases screening during preconception period in Manna District, Southwest Ethiopia: implication for wellness check-up. BMC Health Services Research (2021) 21:56
64. Fira Abamecha, Morankar Sudhakar, Lakew Abebe, Yohannes Kebede, Guda Alemayehu & **Zewdie Birhanu**. Effectiveness of the school-based social and behaviour change communication interventions on insecticide-treated nets utilization among primary school children in rural Ethiopia: a controlled quasi-experimental design. Malar J 20, 41 (2021). https://doi.org/10.1186/s12936-020-03578-x
65. Kiddus Yitbarek, **Zewdie Birhanu,** Gudina Terefe Tucho, Susan Anand, Liyew Agenagnew, Gutema Ahmed Snr, Masrie Getnet, Yonas Tesfaye. **Barriers and Facilitators for Implementing Mental Health Services into the Ethiopian Health Extension Program: A Qualitative Study**. Risk Management and Healthcare Policy 2021:14
66. Tesfaye Y, Agenagnew L, Anand S, Tucho GT, **Birhanu Z**, Ahmed G, Getnet M and Yitbarek K. **Mood Symptoms, Suicide, and Associated Factors Among Jimma Community. A Cross-Sectional Study. Front. Psychiatry** , 2021, 12:640575. doi: 10.3389/fpsyt.2021.640575
67. Aschale A, Fufa D, Kekeba T, **Birhanu Z.** Intention to voluntary blood donation among private higher education students, Jimma town, Oromia, Ethiopia: Application of the theory of planned behaviour. PLoS ONE(2021) 16(3): e0247040. https://doi.org/10.1371/journal.pone.0247040
68. Argaw Ambelu1*, Zewdie Birhanu2, Yimenu Yitayih3, Yohannes Kebede1, Mohammed Mecha4, Jemal Abafita5, Ashenafi Belay6 and Diriba Fufa. Psychological distress during the COVID-19 pandemic in Ethiopia: an online cross-sectional study to identify the need for equal attention of intervention. Ann Gen Psychiatry (2021) 20:22, https://doi.org/10.1186/s12991-021-00344-4
69. Zewdie Birhanu1*, Argaw Ambelu2, Abraraw Tesfaye3, Negalign Berhanu4, Wondwossen Kassahun5, Teferi Daba3, Kifle Woldemichae Prevalence of household food insecurity and associated factors in drought-prone pastoralist communities in Borana, Oromia, Ethiopia. Ethiop. J. Health Dev. 2021; 35(1):00-00
70. Woubishet Girma, Dessalegn Tamiru, Mirkuzie Woldie, Ayantu Kebede, Zewdie Birhanu, Biniam Getachew, Misra Abdulahi, Garumma Tolu Feyissa. Exploring the experience and challenges of maternity waiting homes in Jimma, southwest Ethiopia. African Journal of Midwifery and Women's Health 3 Mar 2021; Vol. 15, No. 1https://doi.org/10.12968/ajmw.2020.0007
71. Birhanu Gutu , Genene Legese ,Nigussie Fikadu , Birhanu Kumela , Firafan Shuma , Wakgari Mosisa , Zelalem Regassa , Yoseph Shiferaw , Lata Tesfaye , Buli Yohannes , Kogila Palanimuthu , Zewdie Birhanu , Desalegn Shiferaw. **Assessment of preventive behavior and associated factors towards. COVID-19 in Qellam Wallaga Zone, Oromia, Ethiopia: A community-based cross-sectional study**. PLoSONE 16(4):e0251062.https://doi.org/10.1371/journal.pone.0251062
72. Zewdie Birhanu1*, Argaw Ambelu1,2, Diriba Fufa3, Mohammed Mecha1,4, Ahmed Zeynudin5, Jemal Abafita6,Ashenafi Belay7, Feleke Doyore8, Lemessa Oljira9, Endale Bacha10, Jilcha Feyisa11, Zinabu Hadis12, Ketema Ayele13,Yohannes Addisu14, Birhanu Gutu15, Demu Tesfaye16, Temesgen Tilahun1,17, Gudeta

Imana18, Tadele Tolosa2,19,Seblework Mekonen2, Yimenu Yitayih20, Nega Jibat21, Mathewos Moges2,22, Ayinengida Adamu2,23,Abraham Teym24, Adamu Kenea25, Taffere Addis26, Akalework Mengesha27and Yohannes Kebede: Risk perceptions and attitudinal responsesto COVID-19 pandemic: an online survey in Ethiopia. Birhanuet al. BMC Public Health (2021) 21:981 https://doi.org/10.1186/s12889-021-10939-x

73. Fira Abamecha, Morankar Sudhakar, Lakew Abebe, Yohannes Kebede, Guda Alemayehu & **Zewdie Birhanu**Perceived sustainability of the school-based social and behavior change communication (SBCC) approach on malaria prevention in rural Ethiopia: Stakeholders' perspectives": Abamechaet al. BMC Public Health (2021) 21:1171 https://doi.org/10.1186/s12889-021-11216-7
74. Mulugeta Dile, Zewdie Birhanu,Gurmessa Tura Perception and Experiences of Sexual Harassment among Women Working in Hospitality Workplaces of Bahir Dar city, Northwest Ethiopia: A qualitative study, BMC public health, BMC Public Health (2021) 21:1119. https://doi.org/10.1186/s12889-021-11173-1
75. Roland Bamou , Martin Rono, Teshome Degefa , Janet Midega, Charles Mbogo, Prophet Ingosi , Alice Kamau , Argaw Ambelu , Zewdie Birhanu , Kora Tushune, Edmond Kopya, Parfait Awono-Ambene, Timoléon Tchuinkam, Flobert Njiokou , Delenasaw Yewhalaw, Christophe Antonio Nkondjio , Joseph Mwangangi. Entomological and Anthropological Factors Contributing to Persistent Malaria Transmission in Kenya, Ethiopia, and Cameroon; J Infect Dis . 2021 Apr 27;223(Supplement_2):S155-S170. doi: 10.1093/infdis/jiaa774.
76. Desalegn Dabaro, Zewdie Birhanu, Abiyot Negash, Dawit Hawaria & Delenasaw Yewhalaw. Effects of rainfall, temperature and topography on malaria incidence in elimination targeted district of Ethiopia. *Malar J* **20**, 104 (2021). https://doi.org/10.1186/s12936-021-03641-1
77. Feleke Doyore Agide, Gholamreza Garmaroudi, Roya Sadeghi, Elham Shakibazadeh, Mehdi Yaseri, Zewdie Birhanu Koricha.Likelihood of Breast Screening Uptake among Reproductive-age Women in Ethiopia: A Baseline Survey for Randomized Controlled Trial.Ethiop J Health Sci.2019;29(5):577.doi:http://dx.doi.org/

Scientific article submitted/under review/accepted

1. [1]**Zewdie Birhanu* (PhD)**, [1]Fira Abamecha (MPH), [3]Nimona Berhanu (B.Pharm), [4]Tadesse Dukessa (MD), [2]Mesfin Beharu (MSc) , [5]Shimellis Legesse (MPH), [1]Yohannes Kebede (MPH) **Perceived patients' healthcare rights during health facility visits in Oromia, Ethiopia: Patients' and health care workers' perspectives; Plose One Journal, 2020**
2. **Zewdie Birhanu*** (PhD), [1]Yohannes Kebede (MPH), [2]Nimona Birhanu (B.Pharm), [3]Mesfin Beharu (MSc), [4]Tadesse Dukessa (MD), [5]Damtew Feyissa (MPH), [1]Chali Endalew (MPH) , [6]Shimellis Legesse (MPH), [1]Demuma Amdisa (MPH, [1]Fira Abamecha (MPH). **Perceived patients' responsibilities during healthcare seeking and medical consultations in Oromia, Ethiopia: A qualitative study; Journal of Preventive Medicine reports, 2020**
3. [1]**Zewdie Birhanu***(PhD), [2]Argaw Ambelu(PhD), [3]Diriba Fufa(MD), [4]Mohammed Mecha(MD), [5]Ahmed Zeynudin(PhD), [6]Jemal Abafita(PhD), [7]Ashenafi Belay (PhD), [8]Feleke Doyore(PhD),[9]Lemessa Oljira(PhD), [10]Endale Bacha(MPH), [11]Jilcha Diribi(MD), [12]Zinabu Hadis (MPH), [13]Ketema Ayele(MPH), [14]Yohannes Addisu(MPH), [15]Birhanu Gutu (MPH), [16]Demu Tesfaye (MD), [17]Temesgen Tilahun (MD), [18]Gudeta Imana (MD), [19]Tadele Tolosa (PhD), [2]Seblework Mekonen (PhD), [20]Yimenu Yitayih (MSc), [21]Nega Jibat(MA), [22]Mathewos Moges (MPH), [23]Ayinengida Adamu(MPH), [24]Abraham Teyim (MPH), [25]Adamu Kenea (MSc), [26]Taffere Addis (PhD), [27]Akalework Mengesha (MA), [1]Yohannes Kebede (MPH). **Risk Perceptions and Attitudinal Responses to COVID-19 Pandemic: An Online Survey in Ethiopia, BMC Public Health, 2020**
4. [1]Diriba Fufa(MD), [2]Yohannes Kebede (MPH), [3]Argaw Ambelu(PhD), [2]**Zewdie Birhanu(PhD)***. **Public knowledge and self-protective behaviors towards COVID-19 in Ethiopia: A cross-sectional study. The Egyptian Journal of Public Health Associations,** 2020
5. [1]Diriba Fufa(MD),[2]Yohannes Kebede (MPH), [3]Argaw Ambelu(PhD), [4]Yohannes Addisu(MPH), [5]Feleke Doyore(PhD), [6]Lemessa Oljira(PhD), [7]Jilcha Diribi(MD), [8]Zinabu Hadis (MPH), [9]Ketema Ayele(MPH), [10]Birhanu Gutu (MPH), [11]Demu Tesfaye (MD), [12]Temesgen Tilahun (MD), [13]Gudeta Imana (MD), [14]Tadele Tolosa (PhD), [3]Seblework Mekonen (PhD), [15]Yimenu Yitayih (MSc), [16]Nega Jibat(MA), [17]Mathewos Moges (MPH), [18]Ayinengida Adamu(MPH),[19]Abraham Teyim (MPH), [20]Taffere Addis (PhD), [2]**Zewdie Birhanu*(PhD)** (corresponding author). **Health care providers' concerns and worries during the COVID-19 pandemic in Ethiopia: An internet based cross-sectional study, Ethiopian Journal of Health Sciences, 2020**
6. *Yohannes Kebede[1*], Abdu Hayder[2], Kassahun Girma[1], Fira Abamecha[1], Guda Alemayehu[3], Lakew Abebe[1], Morankar Sudhakar[1], Zewdie Birhanu[1].* **Primary school students' poetic malaria messages in resource-limited rural endemic settings: A qualitative content analysis, BMC public Health, 2020**
7. Fira Abamecha Ababulgu[1*], , Morankar Sudhakar, Yohannes Kebede, Lakew Abebe, Zewdie Birhanu Koricha. **Effectiveness of school engaged social and behavior change communication on long lasting insecticide treated nets use among rural primary school students in Ethiopia: Propensity score matching analysis approach, BMC Public Health**, 2020
8. **Zewdie Birhanu,** Yohannes Kebede, Gina Chapluae, Kate Dickin. How Health Developmental Armies, Traditional Birth Attendants

And Health Workers Work Together In Pregnancy Care? Linkages, Challenges and Weakness: Qualitative Evidence from Two Districts of Oromia, Ethiopia

9. Zewdie Berhanu[1*], Argaw Ambelu[2], Abraraw Tesfaye[3], Negalign Berhanu[4], Wondwossen Kassahun[5], Teferi Daba[3], Kifle Woldemichael. **Characteristics and Attributes Associated with Households' Food Insecurity in Drought Prone Pastoralist Communities of Southern Ethiopia. Population, nutrition and health journal**

Conference paper

1. Alemseged Abdissa, Tefera Belachew , Zewdie Birhanu · Amare Deribew, Hailemariam Segni · Vivien Tsu Fiona Russell. Ethiopia's Readiness for the Introduction of HPV Vaccine 13th World Congress on Public Health World Health Organization; 04/2012
2. Alemseged Abdissa, Tefera Belachew , Zewdie Birhanu · Amare Deribew, Hailemariam Segni · Vivien Tsu Fiona Russell. Ethiopia's Readiness for the Introduction of HPV Vaccine: Proceedings of the Third Annual Research Conference of Jimma University Organized by Jimma University, January 26-27, 2012. Jimma, Ethiopia.
3. Zewdie Birhanu, [2]Argaw Ambelu, [3]Negalign Berhanu, Abraraw Tesfaye,[5]KyLuu, [5]Deborah Elzie,[6]Roy William Mayega, [6]William Bazeyo, Apollo M. Nkwake, [7]Kifle Woldemichael: Understanding Resilience Dimensions and Adaptive Strategies to the Impact of Recurrent Droughts in Borana Zone, Ethiopia 26-28 October 2015, DJIBOUTI.
4. Zewdie Birhanu*, Gemechis Etana,MorankarSudhakar,LakewAbebe, Guda Alemayehu. School Communities and Religious Leaders as a Change Agents in Malaria SBCC: Experience from USAID-Jimma University Malaria Project. Experience from USAID –JU – ACP Malaria Project in Ethiopia. International SBCC Submit 2016; elevating the science and art of social and behavioral change communication: 8th-10th February, 2016, United Nation Conference Center, Addis Ababa.
5. Zewdie Birhanu*, Gemechis Etana, Morankar Sudhakar,Lakew Abebe, Guda Alemayehu. Barrier to Long Lasting Insecticide treated net (LLIN) Utilization Among the Trained Resource Persons in Community: Experience from USAID –JU – ACP Malaria Project in Ethiopia. International SBCC Submit 2016; elevating the science and art of social and behavioral change communication: 8th-10th February, 2016, United Nation Conference Center, Addis Ababa.
6. Zewdie Birhanu1, Lakew Abebe1, Morankar Sudhakar1, Gunawardena Dissanayake2, Guda Alemayehu2, Gemechis Etana1. EFFECT OF HEALTH EDUCATION FOCUSING ON SCHOOL COMMUNITIES AND RELIGIOUS LEADERS ON COMMUNITY'S MALARIA PREVENTION BEHAVIORS, JIMMA ZONE. A RESULT FROM LOTS QUALITY ASSURANCE SURVEY. 7th malaria research network symposium, Jimma University, march 8-9, 2016 (poster)
7. Zewdie Birhanu, Lakew Abebe, Morankar Sudhakar, Gunawardena Dissanayake, Yemane Ye-ebiyo Yihdego, Guda Alemayehu, Delenasaw Yewhalaw . Malaria Related Perceptions, Seeking Care after Onset of Fever and Anti-Malarial Drug Use in Malaria Endemic Settings of South West Ethiopia. 7th malaria research network symposium, Jimma University, march 8-9, 2016
8. Integrating Strategies for the Prevention of Preeclampsia and Anemia into Community-Based Programs in Ethiopia: A Formative Assessment Result. Zewdie Birhanu, Yohannes Kebede, Stephanie Martin, Gina Chapleau, Kate Dickin. Abstract book of 26th EPHA annual conference; 26-28, Feb 2015. Pp 80
9. Zewdie Birhanu, Lakew Abebe (MPH), Morankar Sudhakar (PhD), Guda Alemayehu (MPH)Access to and Use Gaps of Insecticide-Treated Nets in Jimma Zone: Baseline Result from school and Faith Based Malaria Education Program: Implication for Behavioral Change Communication: Abstract book of 26th EPHA annual conference; 26-28, Feb 2015. Pp 151
10. Zewdie Birhanu |PhD|, Yohannes Kebede |MPH|, Lakew Abebe |MPH|, Guda Alemayehu |MPH|, Morankar Sudhakar |PhD| . School Communities as Social and Behavior Change Communication (SBCC) Agent for Prompting Malaria Preventive Behaviors: Evidence from Ethiopia, 116-20 April 2018, Bali, Indonesia.
11. 10th malaria research network symposium, "Caretakers understanding of malaria, ITN use and care seeking behavior for febrile illness of their children in Ethiopia, 19-20, Dec 2018, Addis Ababa, as presenter
12. Workshop on dissemination of research findings, international institute for primary health care in Ethiopia, "social cultural beliefs, values and norms affecting use of health facility birth in Ethiopia (co-presenter), 21 Dec 2018, Addis Ababa
13. WHO/UNICEF consultative workshop on EPI implementation/embedded research on immunization in Ethiopia; as participant, 12 Dec 2018, Addis Ababa

Modules/Books

1. Abdulnaser A, Binyam A, **Zewdie B**, Tom Heller, Atsede K, Dejene T. (2010). **Health Education, Advocacy and Community Mobilisation Part I: Blended Learning Modules for Health Extension Program**. Federal Ministry of Health, Ethiopia and Open University. December, 2010. Available at www.moh.gov.et; or at www.open.ac.uk/africa/heat
2. Abdulnaser A, Binyam A, **Zewdie B**, Tom Heller, Atsede K, Dejene T. (2010). **Health Education, Advocacy and Community Mobilisation Part II: Blended Learning Modules for Health Extension Program**. Federal Ministry of Health, Ethiopia and Open University. December, 2010. Available at www.moh.gov.et; or at www.open.ac.uk/africa/heat
3. **Zewdie Birhanu**, Dejene Tilahun, Eshetu Girma. **Health communication theories and practice. Distance on module for Health education and promotion students, Jimma University**: June, 2010
4. **Zewdie Birhanu,** Eshetu Girma, Mulgeta Cheka. **Introduction to health education: Distance module for environmental health students**, Jimma University, 2007

Reports

1. Zewdie Birhanu, Negalign Berhanu, Argaw Ambelu. Rapid Appraisal of Resilience to the Effects of Recurrent Droughts in Borana Zone, Southern Ethiopia. Available from: http://www.ranlab.org/wp-content/uploads/2013/11/RAN_Ethiopia_Report_8-July-2015.pdf

2. Zewdie B (PhD), Lakew A (MPH), Morankar S(PhD), Yohannes K(MPH), TSh, Guda A(MPH). School Communities and Religious Leaders as Change Agent for Prompting Malaria Preventive Behaviors: Evidence from SBCC Interventions in Jimma Communities; end line study, 2017
3. Zewdie B (PhD), Lakew A (MPH), Morankar S(PhD), Yohannes K(MPH), TSh, Guda A(MPH). . Knowledge, Attitude and Malaria prevention practices among households in selected districts of Jimma Zone: A baseline result, USAID|Ethiopia, 2013
4. Zewdie Birhanu, Lakew Abebe, Gemechis Itana, MPH, Morankar Sudhakar, PhD. Application of Lots Quality Assurance Survey (LQAS) to Monitor Household Behavioral Practices in Response to Malaria Educations Interventions via School Students and Religious Leaders, 2016
5. **Morankar, Zewdie Birhanu:** IN THEIR OWN WORDS: UNDERSTANDING BARRIERS TO SEXUAL AND REPRODUCTIVE HEALTH ACCESS FOR INTERNALLY DISPLACED YOUNG PEOPLE : A CASE STUDY FROM OROMIA, ETHIOPIA**https://pai.org/resources/in-their-own-words/**

Regular reviewer for journals

1. Ethiopian Journal of Health Sciences
2. Ethiopian Journals of Health Development
3. BMC Public Health
4. Malaria Journal
5. BMC health service journal
6. PloseI Journal

Guest lecturer: Health Education and Promotion

1. Wollega University, 2014, 2015
2. Aksum University, 2016
3. Arsi University, 2016
4. Wachemo University, 2019

External examiner for MSc/MPH thesis

1. Aris University, 2016
2. Ambo University, Nov 2018
3. Wachamo University, Nov 2018
4. Bahir Dar University, 2019

Experience sharing/exchange visit

1. South African medical research center. Feb 27-28, 2018

Training

- Offered advanced qualitative training to Wachemo Univeristy staffs, April 2019
- Advanced qualitative research to JU staffs (2017)
- Advanced qualitative research to JU_JUCAN project (2020)
- Advanced qualitative research to JU_PhD candidacy (2020)

Scientific conference chairing

- Jimma University 9th annual research conference, parallel session chairperson, April 18-19, 2019
- Oromia Physician Association (OPA), electoral committee member, Jan 2021

Research Grants

1. The Effect of Training on use of ITN and community network system on the burden of malaria in vulnerable groups: Cluster randomized trial: A three year project funded by TDR-Project budget: 67,000 USD per year for 3

years (total=200,000 USD). I was Co-PI for this project and a Co-author for four publications that were arise from the project work: Mixed method

2. Challenges and Opportunities in Introducing HPV Vaccine with other Adolescent Health Interventions: Health Care System and Socio-cultural Perspective: Jimma and Addis Ababa-Funded by Path International, 2010 **(Co-PI)**. It was qualitative study and the first in its kind in Ethiopia to introduce Human Papiloma Virus Vaccine (HPV) which is a causative agent for cervical cancer. The finding was presented to stakeholders and I published (with research team), one paper as primary author.
3. Mothers' Experiences and Satisfactions with Health Extension Program in Jimma, Ethiopia: A Cross Sectional Study-Funded by Jimma University, 2011/12 **(PI),** the result was published in BMC health service journal**: Mixed method**
4. Social capital and health status among women in Jimma Zone-Funded by Jimma University-2011/2012 (**Co-PI**)
5. Structural modelling of factors determining HIV testing in Jimma University, Funded by Jimma University-2011/2012 (**Co-PI)**
6. Printed IEC/BCC materials production and distribution In Ethiopia, 2009, young research grant (PI): mixed method
7. Advancing Community's Practice on Prevention and Control of Malaria Through Schools and Faith Based Malaria Prevention Approach in Jimma Zone [Co-PI]: This is a three years project financed by USAID-Ethiopia [2013-2016]. The project benefits community members in three districts of Jimma zone, Oromiya and expected play crucial role in supporting the government effort to reduce malaria burden and assist the moving away towards malaria eradication and elimination of the national plan. In this project, I am a technical team leader and mainly responsible to technical aspect of the program such as communication messages and materials development, testing and dissemination, and monitoring and evaluation as well.
8. Quantifying malaria endemicity in Ethiopia: Implication for moving away from control to elimination (under way), funded byWHO/TDR
9. Understanding residual transmission for sustainable malaria control and enhancement of elimination efforts in Africa; funded by WHO [Delenasaw Yewhalaw, Merkuzie Woldie, Argaw Ambelu, Kora Tushune, Zewdie Birhanu]:
10. Development and Validation of Patient Education Scales and Empowerment Framework in South Western Ethiopia: Efforts to Translate Patient Education and Communication Theories into Practice, 2018: Funded by Jimma University 2018/2019, 230 ETB [PI]
11. Understanding Barriers to Sexual and Reproductive Health Access for Internally Displaced Adolescents and Youth in Ethiopia, Zewdie Birhanu (PhD, MPH| Associate professor) and Morankar Sudhakar (PhD, MSc| Professor); June 2019-funded by PAI $20,000
12. Zewdie Birhanu (PhD, MPH| Associate professor) and Morankar Sudhakar (PhD, MSc| Professor); Oct 2019-Funded by WHO alliance for Health Policy and System researches funded by PAI $30,000: Assessment of Health Policy and Systems Research (HPSR) capacities: Generating and using evidence for policies in Ethiopia
13. Effectiveness of M-health approach to promote MNCH service uptake in Jimma Zone, funded by JU (280,000ETB), Co-PI
14. Evaluating the effectiveness, acceptability, and Feasibility of fully integrated two Mass Drug Administrations and three other complementary health interventions campaigns (2MDA+3): A pragmatic implementation research in Ethiopia; PI (150,000USD)-April 2021 to July 2022.

15. **Identification , synthesis and translation of good practices and evidence for engaging communities in research and social innovation in health care delivery for infectious diseases of poverty in sub-Saharan Africa (PI)-funded by WHO/TDR, May 2021;**
16. **C**OVID-19 and PHC response in Ethiopia, supported by PRIMASYS/WHO-2021

Consultancy services

1. Preparation of distance learning materials for health extension workers in Ethiopia; Health Education, Advocacy and community mobilization part I and Part II: , Ministry of Health, 2010. I was contributed my expertise in the development of this learning material, which is now being used at national level to upgrade health extension workers to level IV. On the top of the materials are freely available for other countries that extend the contribution beyond national level
2. HIV/AIDS and Gender Based Violence project Among flowers plantation workers in Oromia region, Ethiopia-funded by Oromia Regional HAPCO , 2011 (**Co-PI): Mixed method**
3. Assessment of National community mobilization strategies for OTP in Ethiopia, funded by Concern Ethiopia, 2009 (Co-PI): completed: **Qualitative method**
4. Behavioural, Socio-economic and Health Services Determinants of Immunization Service Utilization: A national community and Facility-Based Study in Ethiopia: Funded by WHO and UICEF, 2012 **(Co-PI), completed: Mixed method**
5. Evaluation of community based HIV/AIDS care and support in Ethiopia, FHI international 2011 (Co-PI)
6. Sexual and Reproductive Health services utilization and social empowerment of young people in Western Oromia: A Baseline Formative survey: Funded by Oromiya Development Association[ODA]-completed (Co-PI): Mixed method
7. Resilience African Network: Rapid Appraisal of Resilience to the Effects of Droughts in Borana Zone, Southern Ethiopia [Qualitative researchers core team leader)
8. Integrating strategies for the prevention of preeclampsia and anemia into community-based programs in Ethiopia and Kenya:Trail of Improved Practices (TIPs). A qualitative method: I am a local PI for this project. This study is the first in its kind in the world and aimed to initiate calcium supplementation during pregnancy and being undertaken in collaboration with Cornell University, and funded by MI, Ottawa. In this service, conducted formative assessment as preparatory part of trial study, undertaken cognitive testing for social support scale and implemented TIPs study and participated in development of communication materials for trail counselling, flip chart, and reminder cards.
9. Evaluation of Community based Maternal and new-born care in Afar pastoralist areas, December 2015, Co-investigator ; funded by MI|Ethiopia
10. Maternal and new-born care promotion campaign, funded by KOICA (served as team leader)-key activities included communication material development and testing, campaign design and implementation.

Participation as field researcher, supervisor and facilitator in qualitative research studies

1. TB and HIV project-conducted by Jimma University funded by VILR Project in Gelgel gibe field research center, Ethiopia
2. Evaluation of reproductive Health program, ODA and Tulen University, July, 2009, Ethiopia
3. Risky sexual behaviors among university students (Jimma University students) which was conducted by Jimma University in collaboration National HIV/AIDS prevention and control office, February, 2010 , Ethiopia
4. Home Management of Malaria (HMM) project in Jimma town, Ethiopia, April 2010.
5. Evaluation of BPR implementation in Jimma University specialized hospital, February 2011
6. Evaluation of care and support program for PLHWA in Ethiopia 2012; field researcher

Participation in University and social Affairs

- Served as ethical review committee member at college of public health and medical sciences
- Played significant role to realize and won **NORHED project: A strategic capacity development in Ethiopia and Africa**
- Served as various committee members at department level
- Served as reviewer for University CBE thematic studies
- Active participation in CBE (as supervisor, team leader)
- Head of department, health, behaviour and society
- Developed PhD curriculum-PhD in evidence based health care

Master thesis mentoring

- Advised 60 master thesis and successfully graduated, 90% as primary supervisor
- Supervising 7 PhD as primary supervisor
- Supervising 7 PhD secondary supervisor

Reference 1<br>Name and address	Professor Morankar Sudhakar (PhD)<br>Jimma University, Ethiopia<br>Phone: +0917763778<br>e-mail: morankarsn@yahoo.com
Reference 2<br>Name and address	Professor Mirkuizie Woldie<br>Email: mirkuzie@yahoo.com<br>Phone:+251917804051
Reference 3:<br>Name and address	Professor Kate Dickin (PhD)<br>Cornel University, Nutrition divisions, USA<br>E-mail: kld12@cornell.edu
