# Supplementary material for: Patients’ healthcare, education, engagement, and empowerment rights’ framework: Patients’, caretakers’ and health care workers’ perspectives from Oromia, Ethiopia
Source: PLoS One. 2021 Aug 12;16(8):e0255390. doi: 10.1371/journal.pone.0255390 (PMC8360507; doi:10.1371/journal.pone.0255390)
Supplement: S2 Appendix — (PDF) [file pone.0255390.s002.pdf]

Curriculum Vitae: Dr.Yohannes Kebede

Personal information			
First name(s) / Surname(s)	**Yohannes Kebede Lemu (Ph.D, MPH)**		
Place of Birth	Bedele Town, Buno Bedele Zone, Oromia regional state, Ethiopia		
Current Address(es)	Jimma town, Oromia region , Ethiopia		
Telephone(s)	Mobile	+251913232040	+251961891139
	Office	+251471114439	
E-mail	yohanneskbd@gmail.com or Yohannes.kebede@ju.edu.et		
P.O. Box	378, Jimma, Ethiopia		
Nationality	Ethiopian		
Date of birth	27/04/ 1986		
Gender	Male		

Work experience and position		
Position and ranks	July 2007- Sep 2008	Graduate assistant, Jimma University
	July 2011- September 2014	Lecturer of Health Education and Promotion, Jimma University
	October 16, 2014-June 4, 2019	Assistant professor of health education & promotion Jimma University
	June 5, 2019 onwards	Associate professor of health education & promotion, at department of health, behaviour and Society, Jimma University
Current position held/rank	Ph.D., Associate professor, Department of Health, Behaviour and Society, Institute of Health, Jimma University. His PhD is public health in Health behaviour and communication	
Main activities and responsibilities	Teach undergraduate, distance learners and graduate students various courses (healthy behaviour, health promotion, health communication, health education, health promotion ethics, qualitative research, strategic communication, statistical analysis in behavioural studies)<br>Participate in field works, student supervisions during attachments<br>Advised several undergraduate and graduate students during their thesis work<br>Participated in consultancy services<br>Undertaken/participated in research projects<br>Participated in community services<br>Offer/facilitated trainings	
Name and address of employer	Jimma University<br>Kedir Abrahim Yesuf (Human Resource Management Directorate Director)<br>Jimma town, Oromia region, Ethiopia<br>Web: www.ju.edu.et, mail:erca@ju.edu.et<br>Telephone: +251- (0)47-111-2202<br>Employee ID:1014752000	
Name and address of	Jimma University, Oromia, Ethiopia	

organization	
Type of business or sector	Public University

Educational achievements

Degree awarded	Completed with excellent grade	Dates	Principal subjects covered
PhD in Public health/ Health behaviour and communication		As of May 2021	Philosophy of Public Health, Health education and promotion, Media analysis and health behaviour, Strategic Health communication, Behaviour Modification, Global health and health behaviour, Socio-cultural epidemiology, , Genetics and human behaviour, Advanced Biostatistics infectious disease,
MPH degree in Health Education and Promotion	4.00 (Great distinction) / first class	Sep 2009- June 2011	Health promotion, Health education, strategic health communication, health and human behaviours, behavioural models and theories, Biostatistics, epidemiology, reproductive health, health economics, health service management, research methodologies (quantitative and qualitative
BSc degree in Health Education and Promotion	3.83 (great distinction) first class	Sep 2004- June 2007	Health Education, Health promotion, health communication, Public health courses including epidemiology and Biostatistics

Short term trainings

Title of qualification/certificate awarded	Dates	Principal subjects covered	Organized by Name and type of organisation providing training
1. Certificate of successful completion of training on community mobilization	September 22-26, 2009	Community mobilization process and action cycle, planning, implementation, monitoring and management, evaluation of community mobilization process	Jimma University in Collaboration with AfriComNet

2. Certificate of facilitator for Social marketing for Health and Development	21st -25th March 2011	Trained on social marketing planning process for development workers	AfricomNet in collaboration with Jimma University
3. Certificate of successful completion of trainings on communication material development	April 9-20, 2012	Communication material development, design, production, pretesting, P-process	Johns Hopkins University in collaboration with AIDS resource centre of Ethiopia
4. Certificate of attendance on "Systematic review"	22nd November -3rd December 2014	Introduction to evidence based health care and the systematic review of the evidence<br>The appraisal, extraction and pooling of quantitative data from excremental, non-experimental, diagnostic and prognostic studies, The appraisal, extraction and pooling of qualitative data from qualitative studies, narratives, and text from opinion	The JOANNA BRIGGS institute (Australia) in collaboration with Jimma University, Malaria Alert Center
5. Certified in " Human Subject Research Training On Social And Behavioural Basics"	January, 2015 (online)	History and Ethical principles, Defining Research in Human subject, Belmont report and CITI course introduction, Assessing risk, informed consent, privacy and confidentiality, research with children, Research in public elementary and secondary schools, International research, Internet-based research, and conflict of interest in research involving human subject ethics involving human subjects	Collaborative Institutional Training Initiative (Citi Program) @ University of Miami (Online)
6. Certificate of successful completion of	January, 21-25, 2018	Qualitative research methodology: Designs, methods, planning and	Johns Hopkins University

training on qualitative research methodology with ATLAS ti. V.7		conducting qualitative research, data analysis using ATLAS.ti	and Regional Social & behaviour change communication office-Ethiopia
7. Certificate of achievement on “Trial of Improved Practice prenatal calcium supplementation ” project	March, 2015	Cognitive interviewing techniques and research instrument for contextual appropriateness, trained interviewers on antenatal calcium and iron supplementation and supervised interviewers on implementation of two months households trials of improved practices	Cornel University Canada and Micronutrient Initiative-Ethiopia
8. Instructional skills	November 15-20, 2008	Development of course syllabus, Course objective, competency based education, assessment methods, preparation of examination	ONESTAR-AIDS
9. Trained qualitative research designs for operational research program	March, 2018	Qualitative research designs for primary health care challenges of Oromia regional state setting of Ethiopia	Transform PHCU

Personal skills and competences

Mother tongue(s)	Afan Oromo, Amharic					
Other language(s)	English					
Self-assessment		Understanding		Speaking		Writing
European level (*)		Listening	Reading	Spoken interaction	Spoken production	
Language- English		B2 (independent user)	C1 (proficient user)	B2 (independent user)	B2 (independent user)	C2 (proficient user)
Computer skills and competences	Advanced skill on Microsoft word and Microsoft PowerPoint, Moderate skill on Microsoft excel and Microsoft publishers Advanced skill on SPSS statistical data analysis , basic skills of EPIDATA and basic skills of ATLAS-ti qualitative data analysis software					

Community service and engagement (2016-2021)

Served the community including the professional community through different activities. The following are key community services for which certificate or letter of recognition are produced.

1. Served as **advisory council April 2021-September 2021 for Oromia regional health bureau** under the leadership of OPA-Oromia physician association (2021)
2. Contributed in "**Ethiopia-COVID-19 outbreak-public mobilization and creation**" **by serving as radio panelist** delivered through Jimma community radio organized by OHCEA & Jimma University (2021)
3. Contributed as **risk communication and community engager** as a team of response to COVID-19 pandemic as of March to June, 2020 by producing IEC , educating different segments of community, training frontline health workers on performance of risk communication for COVID-19 (2020)
4. Contributed to **development and validation IEC/BCC materials (3 brochures and 4 posters) for MCH** together with KOICA (Korean international cooperation agency) working on improving maternal and child health service utilization in Jimma zone (Nov, 2017-January, 2018)
5. Contributed in d**esigning and implementation community mobilization and communication campaign for improved MCH** together with KOICA (Korean international cooperation agency) working on improving maternal and child health service utilization in Jimma zone (April, 2018- July, 2018)
6. Served as **school-community mobilizer for advancing community's malaria preventive practices** by producing IEC materials, training student peer educators and teachers who finally reach the community through joint effort of Jimma University and USAID-Ethiopia (2017-2020).
7. Contributed as **reviewer of research proposal for COVID-19 research and innovation grant activities** of Jimma University (2021)
8. Contributed as **reviewer of scientific papers for reputable journals** such as PLOSONE, BMC series, Dove press series, among others (2016-2021)
9. Participated **as oral presenter in 10th Annual research conference** of Jimma University and 9th global knowledge exchange network conferences (April 2019)
10. Participated as **oral presenter in 2nd national SBCC summit-Ethiopia** organized by MOH at Addis Ababa (December, 2019)
11. Served **course deliverer and researcher thesis advisor and thesis evaluator for sister Universities** (Arsi, Wachemo, Wolaita-Sodo, Arba-Minch) (2016-2019)

Peer reviewed publications

1. Yohannes Kebede Lemu, Zewdie Birhanu Koricha, Lakew Abebe Gebretsadik , Ameyu Godesso Roro. Predictors of refusal of provider initiated HIV testing among clients visiting adult outpatient departments in Jimma town, Oromia Region, Ethiopia: unmatched case control study. HIV/AIDS - Research and Palliative Care 2012:4 103–115
2. Zewdie Birhanu, Ameyu Godesso, Yohannes Kebede and Mulusew Gerbaba. Mothers' experiences and satisfactions with health extension program in jimma zone, Ethiopia: a cross sectional study. *BMC Health Services Research* 2013, 13:74
3. Birhanu Z. Yohannes K. and Nigist Z., Does Life Satisfaction Correlate with Risky Behaviors? Finding from Ethiopian Higher Education Students. Global Journal of Research and Review; GJRR[1][1][2], 2014
4. Doyore F, Birhanu Z, Kebede Y, Dejene T,Jara D (2013) Are People Controlling the Danger

or Fear for Condom Use as HIV/AIDS Preventive Message? An Evaluative Type of Study Based on Extended Parallel Process Model. J AIDS Clin Res 4: 264.

5. Yamrot Debela, Zewdie Birhanu, Yohannes Kebede, Malaria Related Knowledge and Child to Parent Communication Regarding Prevention and Control of Malaria among Primary School Students in Jimma Zone, South West Ethiopia, American Journal of Health Research. Vol. 2, No. 5, 2014, pp. 284-290. doi: 10.11648/j.ajhr.20140205.20
6. Hordofa G. Yohannes K. Dejene T., HIV preventive behaviors and associated factors among mining workers in Sali traditional gold mining site Bench Maji zone, south west Ethiopia: A cross sectional study. BMC Public Health, 2014, 14:1003. Doi.10.1186/1471-2458-14-1003.
7. Getnet Abebe Molla, Waju Beyene Salgedo2* and Yohannes Kebede Lemu. Prevalence and determinants of work related injuries among small and medium scale industry workers in Bahir Dar Town, north west Ethiopia. BMC. Annals of Occupational and Environmental Medicine. 27 (12). 2015. Open access. https://doi.org/10.1186/s40557-015-0062-3
8. Befkadu Bekele. Zewdie Birhanu. Yohannes Kebede. Megistu M. Koyira. *Intention to HIV Testing Among Pregnant Women, Areka Town, Wolaita Zone, Southern Ethiopia: A Community Based Cross-Sectional Study*. Developing Country Studies. Vol.6, No.4, pp: 70-81, 2016 ISSN 2224-607X (Paper) ISSN 2225-0565 (Online). www.iiste.org.
9. Stephanie L. Martin, Zewdie Birhanu, Moshood O. Omotayo, Yohannes Kebede, Gretel H. Pelto, Rebecca J. Stoltzfus, and Katherine L. Dickin. *“I Can’t Answer What You’re Asking Me. Let Me Go, Please”: Cognitive Interviewing to Assess Social Support Measures in Ethiopia and Kenya*. Field methods. 29 (4), 317-332. DOI: 10.1177/1525822X17703393
10. Simegnew Handebo, Yohannes Kebede & Sudhakar N. Morankar (2018) *Does social connectedness influence risky sexual behaviours? finding from Ethiopian youths*, International Journal of Adolescence and Youth,23:2,145-158, DOI:10.1080/02673843.2017.1306448
11. Handebo S, Nigusie A, Morankar SN, Kebede Y (2018) “*My Mother Understands My Feelings Before I Tell Her ”Social Connectedness among Youth in North Shewa Oromia Region, Central Ethiopia*. J Community Med Health Educ 8: 628. doi:10.4172/2161-0711.1000628
12. Yohannes Kebede, Gemechis Etana, Eshetu Girma. *Model and Non-Model Mothers are Similar over Significant Aspects of Maternal-Child Health Behaviors in Rural Contexts of Central Ethiopia: Diffusing Healthy Behaviors*. Science Journal of Public Health. Vol.7, No. 1, 2019, pp 10-24. Doi. 10.11648/j.sjph.20190701.13
13. Yohannes Kebede. Guta Tola. Ashenafi Habtamu (2019). *Understanding Socio-Psychological, Demographic, Obstetric, Treatment-Status Aspects of Fertility Desire Among Anti-Retroviral Therapy Clients, Dodota District, Oromia, Ethiopia*. BMJ. Epidemiology (Sunnyvale), an Open Acess Journal. Vol 9 (1): 373. Doi: 10.4172/2161-1165.1000373
14. Yohannes Kebede, Fira Abamecha, Chali Endalew, Mamusha Aman, Abraham Tamirat. *User’s satisfaction with Maternity waiting home services in Jimma Zone, Oromia, Ethiopia: Implications for maternal and neonatal health improvement*. Journal of Women’s Healthcare, 8:464. Doi.10.4172-0420.1000464
15. *Lonsako A., Yohannes K., Tegene L., Tigist A., Bayu B., Directly Observed Treatment Short-Course Compliance and Associated factors among adult Tuberculosis cases in Public Health Institutions of Hadiya Zone, Southern Ethiopia. Journal of Infectious disease and immunity, 8 (1). Pp. 1-9, 2016. Doi. 10.5897/JIDI/2016.0157. http://www.academicjournals.org/JIDI*
16. Kebede Y, *Girma E, Etana G (2019) They were Claimed Model Mothers: Do They Really*

Behave Differently? Implications for Maternal and Child Healthy Behavior Diffusion in Rural Contexts of Central EthiopiaThey were Claimed Model Mothers: Prim Health Care 9: 330.

17. *Gizaw, A.T., Amdisa, D. & Lemu, Yohanne.K. Predictors of substance use among Jimma University instructors, Southwest Ethiopia. Subst Abuse Treat Prev Policy 15, 2 (2020).* https://doi.org/10.1186/s13011-019-0248-8
18. *Tareke KG, Lemu Yohannes K, Yidenekal SA, Feyissa GT (2020) Community's perception, experiences and health seeking behavior towards newborn illnesses in Debre Libanos District, North Shoa, Oromia, Ethiopia: Qualitative study. PLoS ONE 15(1): e0227542. https://doi.org/10.1371/ journal.pone.0227542*
19. *Tesfaye L, Lemu Yohannes K, Tareke KG, Chaka M, Feyissa GT (2020) Exploration of barriers and facilitators to household contact tracing of index tuberculosis cases in Anlemo district, Hadiya zone, Southern Ethiopia: Qualitative study. PLoS ONE 15 (5): e0233358.* https://doi.org/10.1371/journal. pone.0233358
20. *Kebede Y, Yitayih Y, Birhanu Z, Mekonen S, Ambelu A (2020) Knowledge, perceptions and preventive practices towards COVID-19 early in the outbreak among Jimma university medical center visitors, Southwest Ethiopia. PLoS ONE 15(5): e0233744. https://doi.org/10.1371/journal. pone.0233744*
21. *Tareke, K.G., Lemu, Yohannes.K. & Feyissa, G.T. Exploration of facilitators of and barriers to the community-based service utilization for newborn possible serious bacterial infection management in Debre Libanos District, Ethiopia: descriptive qualitative study. BMC Pediatr 20, 303 (2020). https://doi.org/10.1186/s12887-020-02211-9*
22. *Kebede Y, Abebe L, Alemayehu G, Sudhakar M, Birhanu Z (2020) School-based social and behavior change communication (SBCC) advances community exposure to malaria messages, acceptance, and preventive practices in Ethiopia: A pre-posttest study. PLoS ONE 15(6): e0235189. https://doi.org/10.1371/journal. pone.0235189*
23. *Teshome F, Kebede Y, Abamecha F, et al. What do women know before getting pregnant? Knowledge of preconception care and associated factors among pregnant women in Mana district, Southwest Ethiopia: a community-based crosssectional study. BMJ Open 2020;10:e035937. doi:10.1136/ bmjopen-2019-035937*
24. *Gina C. Klemm, Zewdie Birhanu, Stephanie E. Ortolano, Yohannes Kebede, Stephanie L. Martin, Girma Mamo and Katherine L. Dickin. Integrating Calcium Into Antenatal Iron-Folic Acid Supplementation in Ethiopia: Women's Experiences, Perceptions of Acceptability, and Strategies to Support Calcium Supplement Adherence. Global Health: Science and Practice August 2020, https://doi.org/10.9745/GHSP-D-20-00008*
25. *Teshome, F., Kebede, Y., Abamecha, F. et al. Why do women not prepare for pregnancy? Exploring women's and health care providers' views on barriers to uptake of preconception care in Mana District, Southwest Ethiopia: a qualitative study. BMC Pregnancy Childbirth 20, 504 (2020). https://doi.org/10.1186/s12884-020-03208-z*
26. *Kebede, Y., Abebe, L., Alemayehu, G. et al. Effectiveness of peer-learning assisted primary school students educating the rural community on insecticide-treated nets utilization in Jimma-zone Ethiopia. Malar J 19, 331 (2020). https://doi.org/10.1186/s12936-020-03401-7*
27. *Kebede Y, Abebe L, Alemayehu G, Sudhakar M, Birhanu Z. Messenger Students' Engagement Scale: Community Perspectives on School-Based Malaria Education in Ethiopia. Health and Social Care in the Community. Vol. 28 (5). DOI:10.1111/hsc.13193*

28. *Kebede Y, Birhanu Z, Fufa D, Yitayih Y, Abafita J, Belay A, et al. (2020) Myths, beliefs, and perceptions about COVID-19 in Ethiopia: A need to address information gaps and enable combating efforts. PLoS ONE 15(11): e0243024. https://doi.org/10.1371/journal.pone.0243024*
29. *Yitayih Y, Lemu YK, Mekonen S, et al. Psychological impact of COVID-19 outbreak among Jimma University Medical Centere visitors in Southwestern Ethiopia: a crosssectional study. BMJ Open 2021;11:e043185. doi:10.1136/ bmjopen-2020-043185*
30. *Gonfa FT, Lemu Yohannes K., Koricha ZB. Predictors of Women's awareness of common non-communicable diseases screening during preconception period in Manna District, Southwest Ethiopia: implication for wellness check-up. BMC Health Services Research (2021) 21:56.* https://doi.org/10.1186/s12913-021-06067-2
31. *Fira Abamecha, Morankar Sudhakar, Lakew Abebe, Yohannes Kebede, Guda Alemayehu, Zewdie Birhanu.* Effectiveness of school based social and behavior change communication interventions in improving use of insecticide treated nets among primary school students in rural Ethiopia: A quasi-experimental design. BMC Malaria Journal. 19 (5)
32. *Getahun Zebrer; Abraham Tamirat Gizaw; Kasahun Girma Tareke; Yohannes Kebede Lemu*. Implementation, Experience, and Challenges of Urban Health Extension Program in Addis Ababa: A Case study from Ethiopia. BMC Public Health
33. Argaw Ambelu[1*] , Zewdie Birhanu[2], Yimenu Yitayih[3], **Yohannes Kebede[1]**, Mohammed Mecha[4], Jemal Abafita[5], Ashenafi Belay[6] and Diriba Fufa[7]. Psychological distress during the COVID‑ 19 pandemic in Ethiopia: an online cross‑ sectional study to identify the need for equal attentionof intervention. BMC Annals of Pyschiatry. 2021
34. Yitayih Y, **Lemu YK**, Mekonen S, et al. Psychological impact of COVID-19 outbreak among Jimma University Medical Centere visitors in Southwestern Ethiopia: a cross-sectional study. BMJ Open 2021;11:e043185. doi:10.1136/bmjopen-2020-043185
35. Birhanu, Z., Ambelu, A., Fufa, D…..**Kebede, Y**. Risk perceptions and attitudinal responses to COVID-19 pandemic: an online survey in Ethiopia. BMC Public Health 21, 981 (2021). https://doi.org/10.1186/s12889-021-10939-x
36. Abamecha, F., Midaksa, G., Sudhakar, M., Abebe, M., **Kebede, Y**., Alemayehu, G., and Birhanu, Z. Perceived sustainability of the school-based social and behavior change communication (SBCC) approach on malaria prevention in rural Ethiopia: stakeholders' perspectives. BMC Public Health 21, 1171 (2021). https://doi.org/10.1186/s12889-021-11216-7

Manuscript under review/development

1. Ramy Abou Ghayda, Keum Hwa Lee, Young Joo Han,…Yohannes K ebede, et al., Global case fatality rate of coronavirus disease 2019 (COVID-19) by continents and income: a meta-analysis
2. *Yohannes Kebede[1*], Abdu Hayder[2], Kassahun Girma[1], Fira Abamecha[1], Guda Alemayehu[3], Lakew Abebe[1], Morankar Sudhakar[1], Zewdie Birhanu[1]*Primary school students' poetic malaria messages in Ethiopia: A qualitative content analysis
3. Kasahun G, Yohannes K, Garumma T. Exploration of barriers to postnatal care service utilization in Debre Libanos District, Ethiopia: A Descriptive Qualitative Study
4. 1Diriba Fufa(MD), 2Yohannes Kebede (MPH), 3Argaw Ambelu(PhD), 2Zewdie Birhanu(PhD). Public knowledge and self-Protective behaviors towards COVID-19 in

Ethiopia: A cross-sectional Study

5. Zewdie Birhanu*(PhD), 1Yohannes Kebede (MPH), 2Argaw Ambelu(PhD), 3Yohannes Addisu(MPH), Diriba Fufa(MD). Health care providers' concerns and worries during the COVID-19 pandemic: A cross-sectional study in Ethiopia
6. Zewdie B., Yohannes K., Nimona B. et al., A need to develop patients' responsibility framework during medical consultations: Patients' and health care providers' perspectives in Oromia, Ethiopia.
7. Perceived patients' rights during health facility visits in Oromia, Ethiopia: Patients' and health care workers' perspectives
8. 1Zewdie Birhanu* (PhD), 1Fira Abamecha (MPH), 3Nimona Berhanu (B.Pharm), 4Tadesse Dukessa (MD), 2Mesfin Beharu (MSc) , 5Shimellis Legesse (MPH), 1Yohannes Kebede (MPH). Perceived patients' rights during health facility visits in Oromia, Ethiopia: Patients' and health care workers' perspectives
9. Mohammed M., Demuma H., Zewdie B., Yohannes K., Characterization of Perceptions Toward Diabetes Mellitus and Self-Care Practice. Among Diabetes Mellitus Patients Visiting Jimma University Medical Center: Application of Extended Parallel Process Model
10. Yohannes Kebede*1, Lakew Abebe1,Guda Alemayehu2, Morankar Sudhakar1, Zewdie Birhanu1. Operational framework for correcting Insecticide Treated Nets (ITNs) access-use errors: Promoting sleeping arrangement concurrent to the existing measures of access use in resource limited setting, Ethiopia. PLOS ONE
11. Yohannes K., Fira A, Challi E. Mamusha A., Abraham T.Validations of satisfaction tools for maternity waiting home in Jimma zone, Ethiopia
12. Yohannes K., Mamusha A., Abraham T., Addisu B. Fira A., A Husbands' Intention to support pregnant women to use maternity waiting homes in resource limited settings of Jimma zone, Ethiopia
13. Yohannes K., Zewdie B., Firanbon T. Development and validation of Information-Education-Communication (IEC) materials for maternal and newborn health in the context of Jimma zone, Ethiopia
14. Zewdie Birhanu, Yohannes Kebede, Gina Chapluae, Kate Dickin. How Health Developmental Armies, Traditional Birth Attendants And Health Workers Work Together In Pregnancy Care? Linkages, Challenges and Weakness: Qualitative Evidence from Two Districts of Oromia, Ethiopia

Conference paper

1. Yohannes Kebede, Lakew Abebe, Guda Alemayehu, Morankar Sudhakar, Zewdie Birhanu. Diversifying Effective Malaria Elimination Approaches: School Based SBCC Modifies Community's Malaria Messages Exposure, Acceptance and Practices, Jimma Zone, Ethiopia. Poster Presentation, 11th Malaria Research Network of Ethiopia Symposium. Debremarkos University. December 17-18, 2019
2. Yohannes Kebede, Lakew Abebe, Guda Alemayehu, Morankar Sudhakar, Zewdie Birhanu. Diversifying Effective Malaria Elimination Approaches: School Based SBCC Modifies Community's Malaria Messages Exposure, Acceptance and Practices, Jimma Zone, Ethiopia. Oral Presentation, 2nd National SBCC Summit, Addis Ababa, Ethiopia. December 9-11, 2019.
3. Yohannes Kebede, Fira Abamecha, Chali Endalew, Mamusha Aman, Abraham Tamirat. "Do they come again?" pregnant women satisfaction with maternity waiting homes in Jimma

Zone. 2nd Maternal and newborn health conference, Federal Ministry of Health, August 24-26, 2019. Addis Ababa, Ethiopia. .

4. Yohannes Kebede, Fira Abamecha, Chali Endalew, Mamusha Aman, Abraham Tamirat. “Do they come again?” pregnant women satisfaction with maternity waiting homes in Jimma Zone. 10th Jimma University and 9th Global knowledge exchange network annual conference. April 8-9, Jimma University, Jimma, Ethiopia.
5. Integrating Strategies for the Prevention of Preeclampsia and Anemia into Community-Based Programs in Ethiopia: A Formative Assessment Result. Zewdie Birhanu, Yohannes Kebede, Stephanie Martin, Gina Chapleau, Kate Dickin. Abstract book of 26th EPHA annual conference; 26-28, Feb 2015. Pp 80
6. Zewdie Birhanu |PhD|, Yohannes Kebede |MPH|, Lakew Abebe |MPH|, Guda Alemayehu |MPH|, Morankar Sudhakar |PhD|. School Communities as Social and Behavior Change Communication (SBCC) Agent for Prompting Malaria Preventive Behaviors: Evidence from Ethiopia, 116-20 April 2018, Bali, Indonesia.

Reports

1. Yohannes K(MPH), Zewdie B (PhD), Lakew A (MPH), Morankar S(PhD), TSh, Guda A(MPH). School Community As Change Agent For Promoting Malaria Preventive Practices In Jimma Communities. End line Assessment Result of Community Based SBCC, 2019
2. Zewdie B (PhD), Lakew A (MPH), Morankar S(PhD), Yohannes K(MPH), TSh, Guda A(MPH). School Communities and Religious Leaders as Change Agent for Prompting Malaria Preventive Behaviors: Evidence from SBCC Interventions in Jimma Communities; end line study, 2017
3. Zewdie B (PhD), Lakew A (MPH), Morankar S(PhD), Yohannes K(MPH), TSh, Guda A(MPH). . Knowledge, Attitude and Malaria prevention practices among households in selected districts of Jimma Zone: A baseline result, USAID|Ethiopia, 2013

Consultancy and research grants:

He participated in more than 14 consultancy and research activity reports. Consulted the following research projects with different responsibilities such research team, CO-PI, and some as PI.

1. A research Team member in “Assessment of National community mobilization strategies for OTP in Ethiopia: Social Policy Change In Under Five Malnutrition Treatment Service Provision” funded by Concern Ethiopia: Jimma University and Concern Ethiopia, 2009
2. A research team member in “Community Comprehensive Awareness on Tuberculosis and HIV/AIDS” In Kebeles Surrounding Gilgel Gibe Dam. CDC project, 2010 (TB and HIV project-conducted by Jimma University funded by VILR Project in Gelgel gibe field research center, Ethiopia)
3. A research team member of a project “Revitalization Of Primary Health Care Services”: a Study In Jimma Zone, 2011
4. Research team in “Formative study For Introduction Of Cervical Cancer Vaccine, Central and Southwest Ethiopia”, November-January, 2011
5. A research assistant (co-investigator) In “Trial for Improved Practices (TIPs) of Calcium Supplementation for Pregnant Women; Multi-phased formative study, Northwest Shewa; Ethiopia”, between August 2013- January 2015: A project in collaboration between Ethiopian Public Health Institute, Micronutrient Nutrient Initiative Ethiopia, and Cornell

University.
6. A research team member in " Profiling The Works Of Ethio-Wetlands Resource Association (EWNRA) On Integration Of Population, Health And Environment/PHE/ In Oromia Region" : a partial fulfillment project for Master's program in Department of International Health at Johns Hopkins School of Public Health, January 2013
7. Team Member of USAID Malaria Project "Advancing Community Practices for Prevention of Malaria, Jimma Zone" ; a field supervisor for quantitative data collection and qualitative data collector and transcriber , January, 2014
8. A Qualitative Research Team Member In "Evaluating Mutisectoral Strategies For Improved Nutrition And Food Security In Ethiopia (Agriculture-Nutrition Panel Survey): Tufts University (USA) through Save the children ENGINE project and Jimma University, March-April, 2014
9. A Qualitative Research team of Cognitive testing of general and specific social support scale during pregnancy in Ethiopia "Trials of Improved Practices (TIPs) of Calcium Supplementation for Pregnant Women, Northwest Shewa; Ethiopia" project above: A project in collaboration between Ethiopian Public Health Institute, Micronutrient Nutrient Initiative Ethiopia, and Cornell University.
10. A Qualitative Research Team Member in "Gender differences in Access and Uptake of nutrition messages in Ethiopia" conducted by a PhD research assistant ENGINE project at Friedman School Of Nutrition Science And Policy, Tufts University (USA) through Save the children ENGINE project in Ethiopia, June 17-July 25, 2015
11. Satisfaction on maternity waiting home services and social supports in prenatal care-funded by Jimma University mega project (2016-18) (PI)
12. Maternal and new-born care promotion campaign, funded by KOICA (served as consultancy team)- key activities included communication material development and testing, campaign design and implementation (2019)
13. Validation and development of patient education and communication framework in public hospitals in Jimma Zone (Co-PI)-funded by Jimma University mega project (2019-2020)
14. Project team member of effectiveness of health campaign in Ethiopia: Implementation study (2021-on going)

**Reference persons**	
Reference 1<br>Name and address	Professor Morankar Sudhakar (PhD)<br>Jimma University, Ethiopia<br>Phone: +251917763778<br>e-mail: morankarsn@yahoo.com
Reference 2<br>Name and address	Zewdie Birhanu (MPH, PhD, Associate professor)<br>Head, department of Health-Behaviour and Society<br>Jimma University<br>Phone: +251917025852<br>E-mail: zbkoricha@yahoo.com
