# Supplementary material for: Patients’ healthcare, education, engagement, and empowerment rights’ framework: Patients’, caretakers’ and health care workers’ perspectives from Oromia, Ethiopia
Source: PLoS One. 2021 Aug 12;16(8):e0255390. doi: 10.1371/journal.pone.0255390 (PMC8360507; doi:10.1371/journal.pone.0255390)
Supplement: S3 Appendix — (PDF) [file pone.0255390.s003.pdf]

atlas.ti report

Families: List of Code Families and their Members

Code Family	Codes
Patient right: Challenges	• patient right challenge: blaming patients for their illness<br>• patient right challenge: fear of denial of services<br>• patient right challenge: how do not teach patients<br>• patient right challenge: insult from HWs<br>• patient right challenge: lack of awareness from patients<br>• patient right challenge: negligence from HWs
Patient right:insuring strategies	• patient right insurance: teach on service delivery methods<br>• patient right insurance: teach patients about their rights
Patient_right: Access to drugs	• Patient right: get drugs<br>• patient right : get drug<br>• patient right : get necessary drugs<br>• patient right: get appropriate dose of drugs<br>• patient right: get drug<br>• Patient right: get medications<br>• patient right: get right drug<br>• Patient right: right to choice drug<br>• patient right: right to get officious drugs<br>• patient right: right to refuse any drug
Patient_right: Access to personal medical records and cards	• patient right: access to own medical card
Patient_right: Admissions	• patient right : right to be admitted<br>• Patient right: get bed for admission
Patient_right: Diagnosis and investigations	• Patient right: access to lab services<br>• patient right : get lab result without delay<br>• Patient right: well informed about service delivery process<br>• patient right: can avoid uncomfortable procedure<br>• Patient right: get lab result quickly<br>• Patient right: get necessary examination<br>• Patient right: say no to give sample if don't want to<br>• Patient right: shouldn't be forced for examination
Patient_right: diet and food advice	• Patient right: get diet advice<br>• patient right: get diet of their preference when admitted<br>• patient right: get food quality-no expired food<br>• patient right: get meal service when admitted
Patient_right: Don't know	• patient right: Don't know<br>• Patient right: right to information-do's-don't

	• right vs responsibility-Don't know
Patient_right: Equal treatment without discriminations	• Patient challenge: discriminate by language
Patient_right: Facility use and instructions or directions to use	• Patient right: get education how to use facilities in hospital<br>• Patient right: right to use toilet<br>• Patient right: well informed about service delivery process<br>• Patient right: get help on directions<br>• Patient right: get help/support
patient_right: have caretaker	• Patient challenge: resting place for caretaker<br>• Patient right: allow movement of caretaker
Patient_right: Infection_Preventions	• Patient right: get mosquito net while admitted<br>• Patient right: get protected from other infections
Patient_right: Know their illness	• Patient right: know my illness<br>• Patient right: right to tell their illness<br>• patient right : get information about their illness<br>• Patient right: understand their illness in their own language
Patient_right: Language use	• language barrier- in diagnosis<br>• language barrier-no translation<br>• Patient challenge: discriminate by language<br>• Patient right: understand their illness in their own language<br>• Patient right: use one's own language
Patient_right: No right	• Patient right: I have no right<br>• patient right: no right-dependent on health worker<br>• Patient right: only beg-no right
Patient_right: Prompt care	• Patient right: promptly get care medication<br>• Patient right: seen on time<br>• patient right : get lab result without delay<br>• patient right: get service promptly<br>• Patient right: get treatment on time
Patient_right: referral and transfer	• Patient right: right to be referred to higher care<br>• Patient right: right to transferred to other hospital
Patient_right: respect and dignity	• Patient right: get respected<br>• patient right: get dignity<br>• patient right: moral respect<br>• patient right: not respected at all<br>• Patient right: no respect by guard<br>• Patient right: Insult
Patient_right: Rest place/rooms	• Patient challenge: resting place for caretaker

Patient_right: Service and treatment	• Patient right: access to lab services<br>• Patient right: enough treatment<br>• Patient right: get services they want<br>• patient right : get free service as stated by law<br>• patient right : get service without financial barriers<br>• patient right : get treatment<br>• Patient right: well informed about service delivery process<br>• patient right: get adequate services<br>• patient right: get any service at any time<br>• patient right: get meal service when admitted<br>• Patient right: get necessary services while stay in hospital<br>• Patient right: get service in their own language/language they can understand<br>• patient right: get service promptly<br>• patient right: get service when admitted<br>• Patient right: get treatment on time<br>• patient right: get treated without discrimination<br>• Patient right: get update on treatment progress
Patient_right: tell history and expression of ideas	• Patient right: right to tell their illness<br>• Patient right: express my feeling
Patient_right: Informed decision and treatment choice	• Patient right: get services they want<br>• patient right: get diet of their preference when admitted<br>• Patient right: right to choice drug<br>• patient right: right to refuse any drug<br>• patient right: right to refuse medications<br>• patient right: right to refuse operations<br>• patient right: right to refuse x-rayed<br>• Patient right: right to select providers<br>• Patient right: right to select operation doctor<br>• Patient right: say no to give sample if don't want
Patient_right: treatment progress monitor	• Challenge: Information on health progress<br>• Patient right: get update on treatment progress
Patient_right: Counseling and information, empowering	• Patient right: get education how to use facilities in hospital<br>• patient right : get counseling<br>• patient right : get education on their health challenges<br>• patient right : get information about their illness<br>• Patient right: well informed about service delivery process<br>• Patient right: get advice on my health<br>• Patient right: get diet advice<br>• Patient right: right to information-do's-don't
Patient_right: Follow up care	• Patient right: right get appointment-follow up care
